# Oncoplastic Breast Consortium consensus conference on nipple-sparing mastectomy

**DOI:** 10.1007/s10549-018-4937-1

**Published:** 2018-09-04

**Authors:** Walter P. Weber, Martin Haug, Christian Kurzeder, Vesna Bjelic-Radisic, Rupert Koller, Roland Reitsamer, Florian Fitzal, Jorge Biazus, Fabricio Brenelli, Cicero Urban, Régis Resende Paulinelli, Jens-Uwe Blohmer, Jörg Heil, Jürgen Hoffmann, Zoltan Matrai, Giuseppe Catanuto, Viviana Galimberti, Oreste Gentilini, Mitchel Barry, Tal Hadar, Tanir M. Allweis, Oded Olsha, Maria João Cardoso, Pedro F. Gouveia, Isabel T. Rubio, Jana de Boniface, Tor Svensjö, Susanne Bucher, Peter Dubsky, Jian Farhadi, Mathias K. Fehr, Ilario Fulco, Ursula Ganz-Blättler, Andreas Günthert, Yves Harder, Nik Hauser, Elisabeth A. Kappos, Michael Knauer, Julia Landin, Robert Mechera, Francesco Meani, Giacomo Montagna, Mathilde Ritter, Ramon Saccilotto, Fabienne D. Schwab, Daniel Steffens, Christoph Tausch, Jasmin Zeindler, Savas D. Soysal, Visnu Lohsiriwat, Tibor Kovacs, Anne Tansley, Lynda Wyld, Laszlo Romics, Mahmoud El-Tamer, Andrea L. Pusic, Virgilio Sacchini, Michael Gnant

**Affiliations:** 1 Breast Center, University Hospital Basel, Spitalstrasse 21, 4031 Basel, Switzerland; 2 University of Basel, Basel, Switzerland; 3 Department of Gynecology, Medical University of Graz, Graz, Austria; 4 Department of Plastic and Reconstructive Surgery, Wilhelminenspital, Vienna, Austria; 5 Breast Center Salzburg, University Clinic Salzburg, Paracelsus Medical University Salzburg, Salzburg, Austria; 6 Department of Surgery and Comprehensive Cancer Center, Medical University of Vienna, Vienna, Austria; 7 Breast Unit, Hospital de Clínicas de Porto Alegre, Porto Alegre, Brazil; 8 Breast Oncology Division, University of Campinas, Campinas, SP Brazil; 9 Positivo University Medical School, Curitiba, Brazil; 10 Gynecology and Breast Unit, Goiás Anticancer Association, Hospital Araújo Jorge, Goiânia, Brazil; 11 Department of Gynecology and Breast Center, Charité University Hospital, Berlin, Germany; 12 Department of Obstetrics and Gynecology, Medical School, University of Heidelberg, Heidelberg, Germany; 13 Breast Center, University Hospital Düsseldorf, Düsseldorf, Germany; 14 Department of Breast and Sarcoma Surgery, National Institute of Oncology, Budapest, Hungary; 15 Multidisciplinary Breast Unit, Azienda Ospedaliera Cannizzaro, Catania, Italy; 16 Division of Breast Surgery, European Institute of Oncology, Milan, Italy; 17 Breast Surgery Unit, San Raffaele Hospital, Milan, Italy; 18 Mater Misericordiae University Hospital, Dublin, Ireland; 19 Breast Health Unit, Shaare Zedek Medical Center, Hebrew University of Jerusalem, Jerusalem, Israel; 20 Department of Surgery, Kaplan Medical Center, Rehovot, Israel; 21 Breast Unit, Champalimaud Clinical Centre, Champalimaud Foundation, Lisbon, Portugal; 22 Breast Surgical Oncology, Clinica Universidad de Navarra, Madrid, Spain; 23 Department of Surgery, Capio St Göran’s Hospital, Stockholm, Sweden; 24 Department of Molecular Medicine and Surgery, Karolinska Institutet, Stockholm, Sweden; 25 Department of Surgery, Central Hospital, Kristianstad, Sweden; 26 Breast Center, Lucerne Cantonal Hospital, Lucerne, Switzerland; 27 Breast Center, Hirslanden Clinic St. Anna, Lucerne, Switzerland; 28 Breast Center Zurich, Zurich, Switzerland; 29 Breast Center Thurgau, Cantonal Hospital Frauenfeld, Frauenfeld, Switzerland; 30 Institute of Sociology, University of St. Gallen, St. Gallen, Switzerland; 31 Department of Gynecology, Lucerne Cantonal Hospital, Lucerne, Switzerland; 32 Department of Plastic, Reconstructive and Aesthetic Surgery, Centro di Senologia della Svizzera Italiana (CSSI), Ospedale Regionale di Lugano, Ente Ospedaliero Cantonale (EOC), Lugano, Switzerland; 33 Breast Center, Hirslanden Clinic Aarau, Aarau, Switzerland; 34 Breast Center St. Gallen, St. Gallen, Switzerland; 35 Department of Obstetrics and Gynecology, Centro di Senologia della Svizzera Italiana (CSSI), Ospedale Regionale di Lugano, Ente Ospedaliero Cantonale (EOC), Lugano, Switzerland; 36 Department of Clinical Research, University Hospital Basel, Basel, Switzerland; 37 Department of Surgery, Faculty of Medicine Siriraj Hospital, Mahidol University, Bangkok, Thailand; 38 Breast Surgery Unit, Guy’s Hospital, Guy’s and St. Thomas’ NHS Foundation Trust, London, UK; 39 Department of Surgery, Royal Liverpool University Hospital, Liverpool, UK; 40 Department of Oncology and Metabolism, University of Sheffield, Sheffield, UK; 41 Department of Surgery, New Victoria Hospital, Glasgow, UK; 42 Department of Surgery, Memorial Sloan Kettering Cancer Center, New York, NY USA; 43 Division of Plastic and Reconstructive Surgery, Brigham and Women’s Hospital, Boston, MA USA; 44 Sektion Senologie, Helios Klinikum Wuppertal GmbH, University Hospital of University Witten/Herdecke, Wuppertal, Germany

**Keywords:** Breast cancer surgery, Nipple-sparing mastectomy, Immediate breast reconstruction

## Abstract

**Purpose:**

Indications for nipple-sparing mastectomy (NSM) have broadened to include the risk reducing setting and locally advanced tumors, which resulted in a dramatic increase in the use of NSM. The Oncoplastic Breast Consortium consensus conference on NSM and immediate reconstruction was held to address a variety of questions in clinical practice and research based on published evidence and expert panel opinion.

**Methods:**

The panel consisted of 44 breast surgeons from 14 countries across four continents with a background in gynecology, general or reconstructive surgery and a practice dedicated to breast cancer, as well as a patient advocate. Panelists presented evidence summaries relating to each topic for debate during the in-person consensus conference. The iterative process in question development, voting, and wording of the recommendations followed the modified Delphi methodology.

**Results:**

Consensus recommendations were reached in 35, majority recommendations in 24, and no recommendations in the remaining 12 questions. The panel acknowledged the need for standardization of various aspects of NSM and immediate reconstruction. It endorsed several oncological contraindications to the preservation of the skin and nipple. Furthermore, it recommended inclusion of patients in prospective registries and routine assessment of patient-reported outcomes. Considerable heterogeneity in breast reconstruction practice became obvious during the conference.

**Conclusions:**

In case of conflicting or missing evidence to guide treatment, the consensus conference revealed substantial disagreement in expert panel opinion, which, among others, supports the need for a randomized trial to evaluate the safest and most efficacious reconstruction techniques.

**Electronic supplementary material:**

The online version of this article (10.1007/s10549-018-4937-1) contains supplementary material, which is available to authorized users.

## Introduction

The emphasis on esthetic outcomes and quality of life (QoL) after breast cancer treatment has motivated surgeons to develop nipple-sparing mastectomy (NSM) and immediate reconstruction. NSM was initially reserved for patients with small tumors, remote from the nipple, based on reports of high rates of nipple involvement in larger tumors [1]. Indications have recently broadened to include both the risk reducing setting and larger tumors resulting in a dramatic increase in the use of NSM [2–6].

NSM reduces the adverse psychological impacts of mastectomy [7]. Two large surveys of breast cancer survivors demonstrated similar satisfaction between mastectomy with reconstruction and breast-conserving surgery (BCS), even though the latter is considered first choice whenever appropriate due to the limited extent of surgery [8, 9]. Preservation of the nipple–areola complex (NAC) improves patients’ post-mastectomy QoL when compared with non-nipple-sparing mastectomies [10, 11].

Even though NSM and immediate reconstruction have been established in routine clinical practice with a supporting evidence base, many questions remain unanswered. The Oncoplastic Breast Consortium (OPBC) consensus conference on NSM was held to address the most urgent questions in clinical practice and research. The goal was to recommend standard surgical approaches pertaining to NSM and reconstruction based on the integration of data from all types of clinical evidence including experience drawn from contemporary practice and innovations in surgery. This report summarizes the consensus recommendations of the panel.

## Methods

### Oncoplastic Breast Consortium

The OPBC is committed to bringing safe and effective oncoplastic breast surgery to routine patient care, namely oncoplastic breast-conserving surgery (OPS), as well as NSM and skin-sparing mastectomy (SSM) with immediate reconstruction [12]. After the first consensus conference on standardization of OPS in German-speaking countries in 2017, the need was recognized for an independent non-profit organization to develop recommendations that are applicable globally [13].

The core of the consortium consists of one coordinator per country who recommends national panelists based on their scientific and clinical record of accomplishment, international reputation, and motivation to support actively the mission of the OPBC. The selection of panelists is driven by evident expertise in breast cancer management with a practice primarily dedicated to breast cancer at regional referral centers. The panel includes specialists from private, public, community, and academic settings. The panel consists predominantly of oncologic and oncoplastic breast surgeons with a background in surgery and gynecology, because they meet the patients first on their treatment path and have the initial discussion about their surgical treatment. Several reconstructive surgeons were included in the panel, which consists of 44 OPBC coordinators and panelists from 14 countries across four continents (see Supplementary Appendix 1).

In addition, the OPBC has a growing membership of surgeons from gynecologic oncology, general surgery, surgical oncology, and reconstructive breast surgery. The OPBC was founded in March 2017 and has recruited 187 members from 46 countries within 1 year, including the 44 coordinators and panelists. The OPBC pursues its mission to continuously improve OPS, NSM and SSM by bringing international experts together to address controversial topics, by offering oncoplastic training courses and by performing relevant clinical research projects.

### Preparation for the consensus conference

The expert panel of the consensus conference consisted of the OPBC coordinators and panelists. Before the conference, the chair provided all panelists with the topics for debate. The pre-defined protocol of the conference was published on the OPBC website and was repeatedly updated until March 05, 2018 [14]. The panelists reviewed the questions for the consensus session. The organizers adjusted the questions according to the feedback by iterative consultation over the months preceding the conference, thereby applying the modified Delphi methodology.

### Consensus conference

The OPBC consensus conference on NSM was held in Basel, Switzerland, on March 15, 2018. During the meeting, panel members presented detailed evidence summaries relating to each topic for debate, followed by an interactive discussion. In the second half, each group of questions was introduced with a short discussion, followed by electronic voting on the entire category of questions, immediate face-to-face discussion of the results, and re-voting if appropriate.

Of the 44 OPBC coordinators and panelists who participated in the development of the set of questions, 38 (86%) attended the conference in person. A patient advocate was invited to the conference and participated in voting. Even though voting was restricted to the panel, all OPBC members were able to join the meeting live online.

For most statements or questions, voting was in the format yes, no or abstain, but for a minority, the single most appropriate answer was selected from the list of options. Abstaining was recommended if panel members had a conflict of interest or felt that the question was not clear or outside of their expertise, or that the correct answer was missing.

### Review

References were identified through searches of PubMed with the search terms “mastectomy, subcutaneous” OR “mastectomy” AND “subcutaneous” OR “subcutaneous mastectomy” OR “nipple” AND “sparing” AND “mastectomy” OR “nipple-sparing mastectomy” from January 2000 until April 2018. Two authors independently considered all original series and reviews during that time period and selectively included additional references cited in those publications. Articles were also identified through searches of the authors’ own files.

### Report

**Fig. 1:**
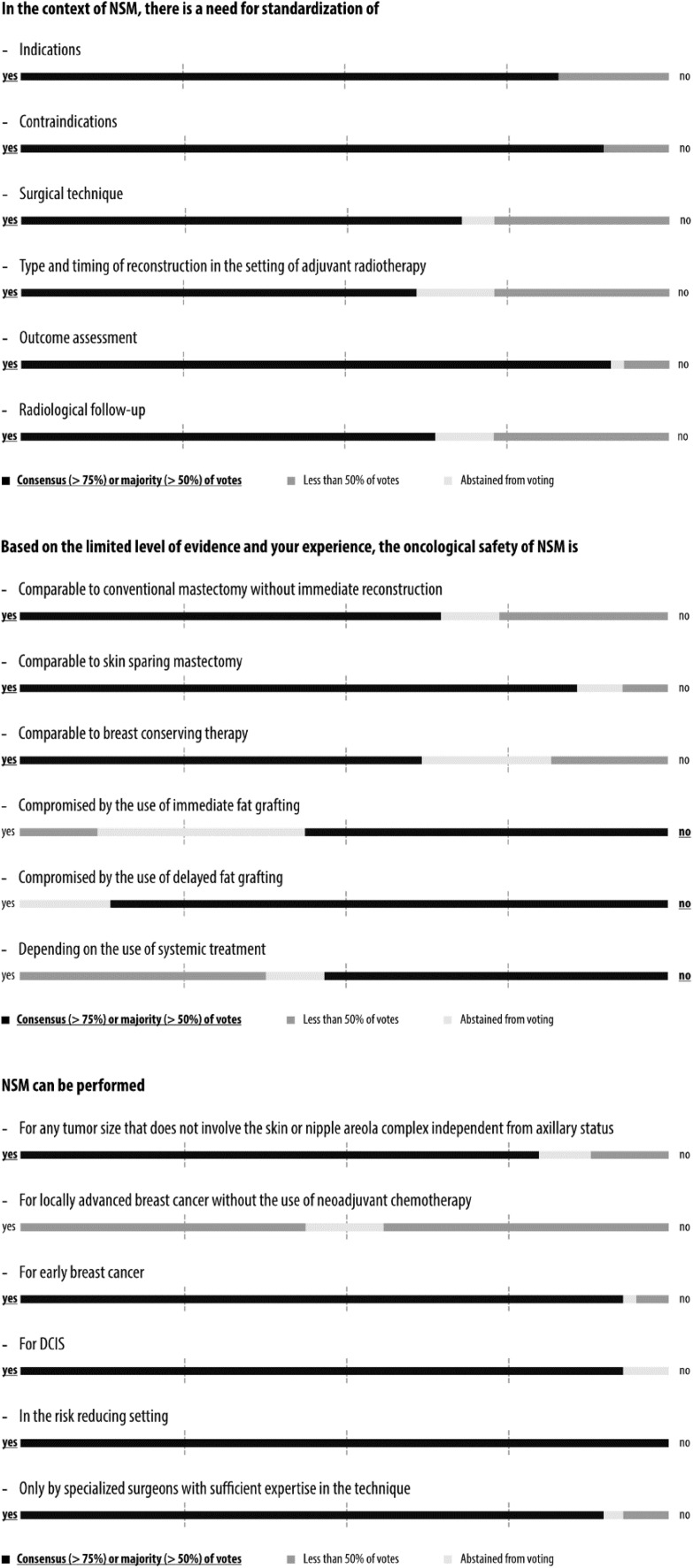
Consensus conference results: standardization, oncological safety and indications

**Fig. 2:**
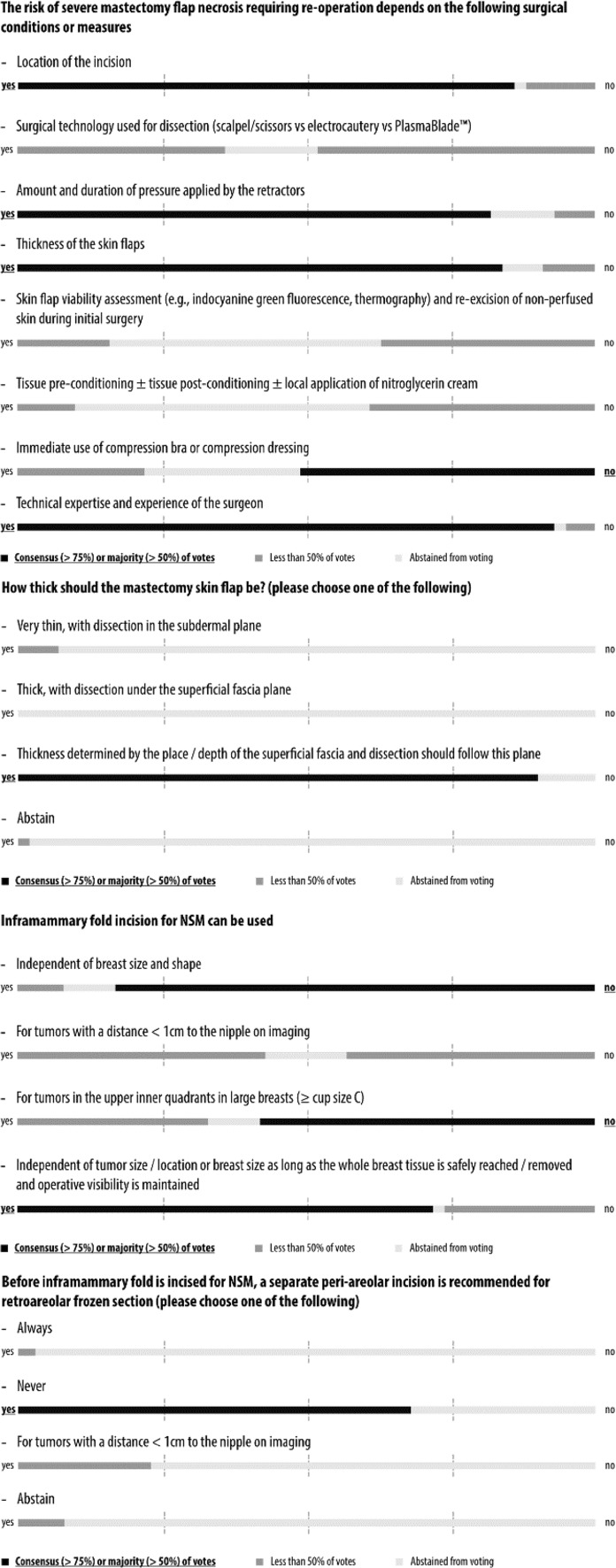
Consensus conference results: surgical technique

**Fig. 3:**
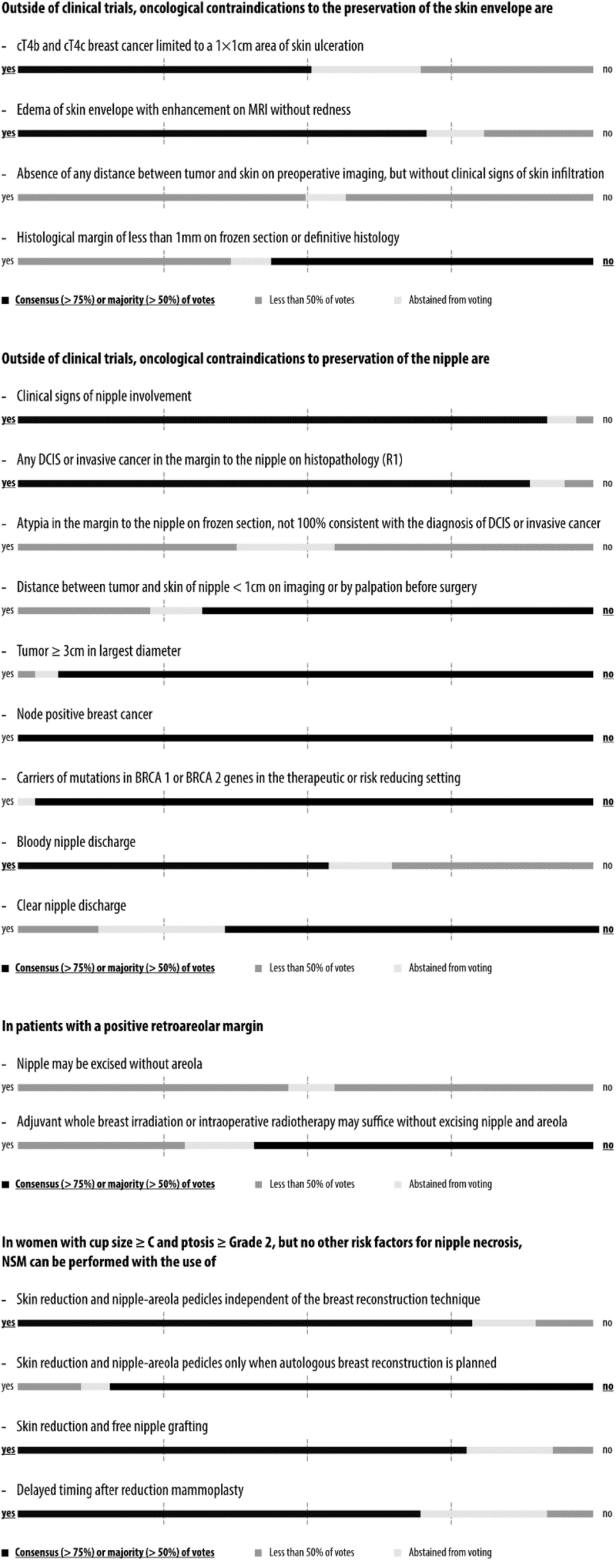
Consensus conference results: contraindications

**Fig. 4:**
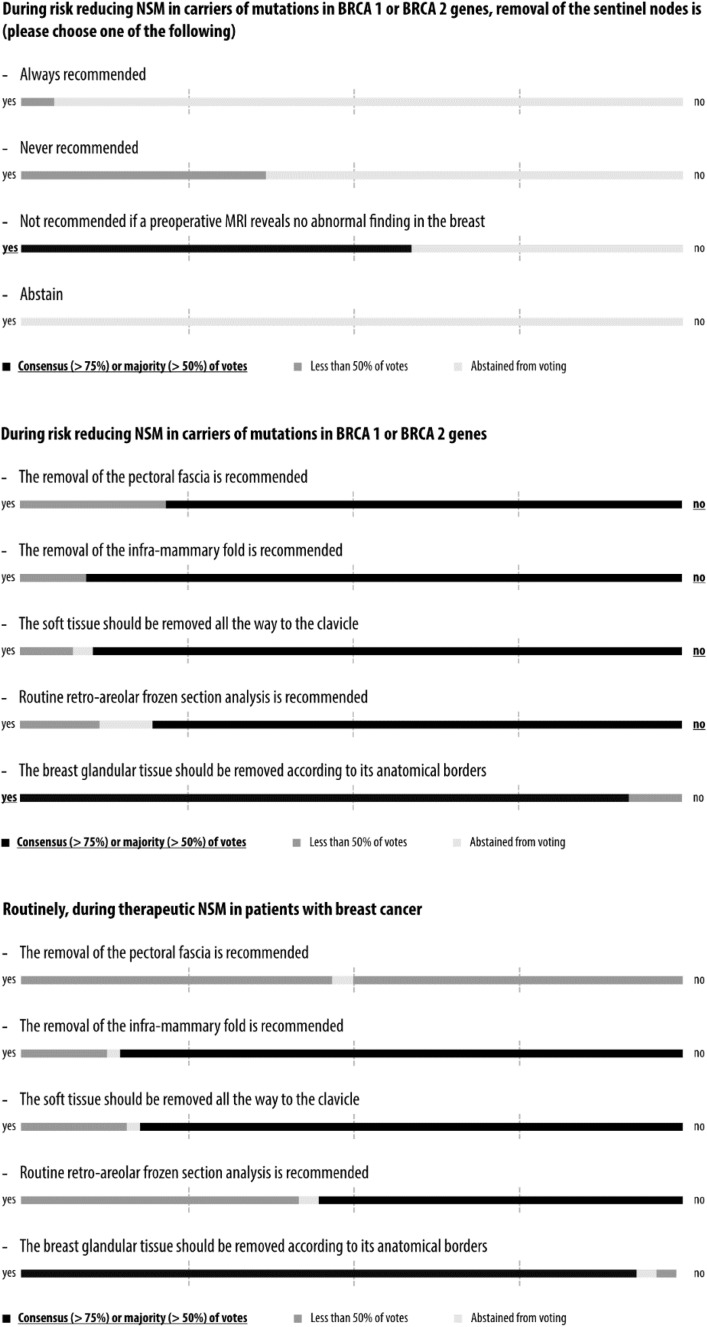
Consensus conference results: special considerations in the risk reducing and therapeutic setting

**Fig. 5:**
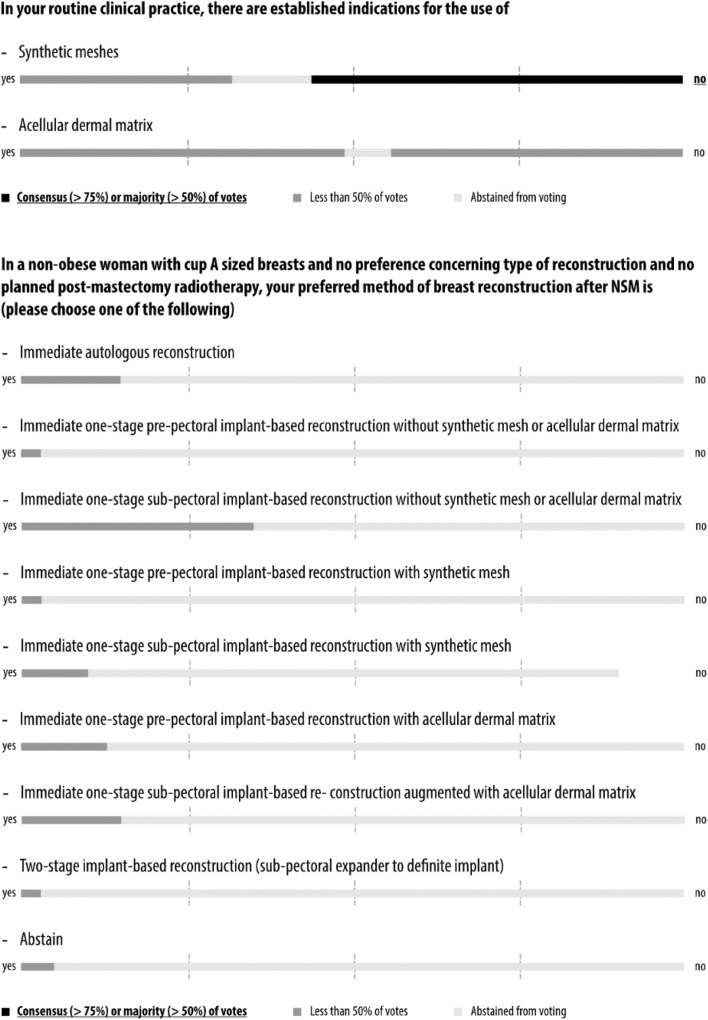
Consensus conference results: breast reconstruction

**Fig. 6:**
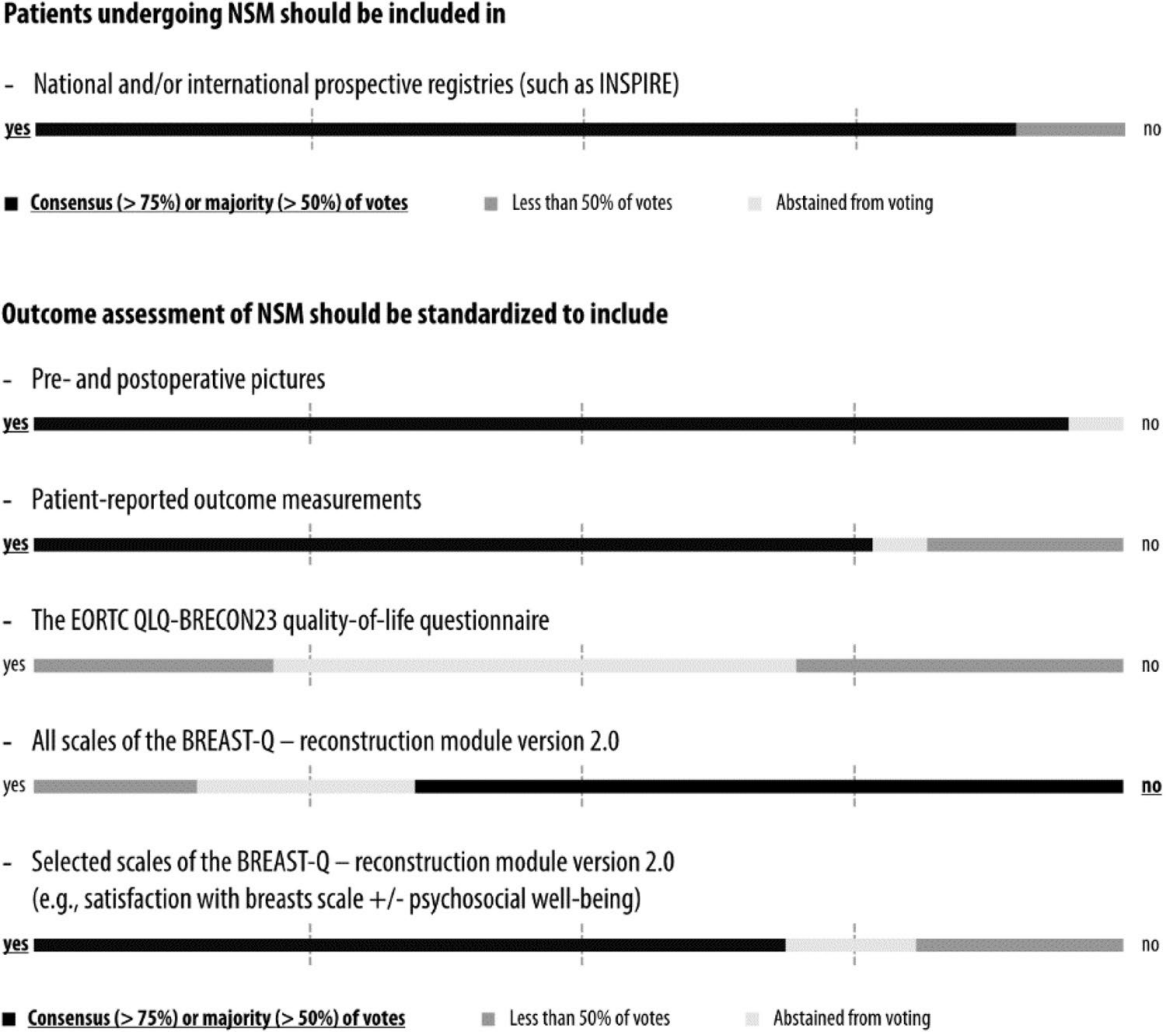
Consensus conference results: registries and outcome assessment

Simple majority was pre-defined by agreement among 51–75% of the panelists and consensus by agreement above 75%. The questions, answers, and discussions were brought into context with current evidence from the literature in the form of this report, which was circulated among all 44 panelists in an iterative process until agreement was reached on each question before publication. The wording conveys the strength of panel support for each recommendation. Voting results are shown graphically in Figs. 1, 2, 3, 4, 5, and 6 and as exact numbers in supplementary appendices [4–9].

## Results

A total of 71 questions in 18 categories covered various aspects of NSM with immediate reconstruction in the risk reducing and therapeutic setting, including oncological considerations, technical indications and contra-indications, and outcome assessment. Consensus recommendations were reached in 35 questions, majority recommendations in 24, and no consensus and no majority in the remaining 12 (Figs. 1, 2, 3, 4, 5, 6 and Supplementary Appendices 4–9). The review of the literature revealed predominantly observational studies, with only two randomized controlled trials (RCTs). In the absence of supporting data from clinical studies, recommendations were based on personal opinion or preference (level III evidence according to the United States Preventive Services Task Force) [15].

### Specific areas in need for standardization

The first set of questions aimed at setting the stage before individually assessing each question in depth in the following categories (Fig. 1 and Supplementary Appendix 4). The panel reached consensus when identifying the need for standardizing indications, contraindications and outcome assessment, while a clear majority recommended standardization of surgical technique, type and timing of reconstruction in the setting of adjuvant radiotherapy and radiological follow-up.

### Oncological safety of NSM

A clear majority of the panel felt that the oncological safety of NSM is comparable to conventional mastectomy without reconstruction and to BCS, and the panel reached consensus that it is comparable to SSM if cases are selected appropriately (Fig. 1 and Supplementary Appendix 4). Residual breast tissue left behind underneath the skin envelope in NSM and SSM has raised concerns about the completeness of mastectomy [16]. The available evidence on oncological safety of NSM is based on observational studies of low overall quality [17]. One of the pioneering prospective single-center studies of 216 NSM patients reported loco-regional recurrence (LRR) rates of 8.5% among irradiated and 28.4% among non-irradiated patients at a median follow-up of 13 years [18]. The high rate of LRR was primarily attributed to the non-radical surgical technique of subcutaneous mastectomy that was used at the time of recruitment between 1988 and 1994. In addition, the patient population included many large, node-positive tumors. However, more than 60 NSM series have been published since 2000; a selection of which is shown in Supplementary Appendix 2. The vast majority were single-center studies. They almost all showed acceptable rates of recurrence after NSM. A recent analysis of the Surveillance, Epidemiology, and End Results (SEER) database identified 2440 breast cancer patients who received NSM from 1998 to 2013. The 5- and 10-year cancer-specific and overall survival rates were 96.9% and 94.9%, and 94.1% and 88.0%, respectively, very much in keeping with standard mastectomy techniques [19].

While a slim majority of the panel, with one-third abstaining, believed that the oncological safety of NSM is not compromised by the use of immediate fat grafting, there was clear consensus that safety is not compromised when timing is delayed (Fig. 1 and Supplementary Appendix 4). The oncological safety of autologous fat grafting after NSM and immediate reconstruction has been widely debated [20]. Fat grafting can improve esthetic outcomes of breast reconstruction when compromised by radiotherapy, for example, which may translate into increased patient satisfaction and psychosocial wellbeing [21, 22]. Preclinical studies, however, indicated that fat grafting may promote breast cancer growth and metastasis [23]. Several reviews and contemporary patient series show that fat grafting does not increase the risk of recurrence when applied as a delayed procedure after autologous reconstruction [24–27]. Finally, a slim majority of the panel felt that the oncological safety of NSM did not depend on the use of systemic therapy.

### Indications for NSM

There was consensus that NSM can be performed for any tumor size that does not involve the skin or nipple areola complex (NAC) independent of axillary status (Fig. 1 and Supplementary Appendix 4). However, the panel was divided when asked if NSM could be offered to patients with locally advanced breast cancer (LABC) without the use of successful neoadjuvant chemotherapy (NACT). Several groups have broadened the indication for NSM to include patients with LABC, who have been successfully down-staged with neoadjuvant systemic therapies [2, 6]. The evidence base for use of NSM in this setting is currently poor and more studies with longer follow-up are required.

The panel recommended NSM for early breast cancer and ductal carcinoma in situ (DCIS), and, unanimously, in the risk reducing setting. This latter indication is now well-established in clinical practice [4, 5, 28].

The panel strongly felt that only specialized surgeons with high-volume training should perform NSM. This claim has been made repeatedly in the past by specialized breast and plastic surgeons, and should be supported by volume-outcome research with caseload as predictor and rate of complications and local recurrence as outcomes. NSM certainly is technically challenging and surgeons experience greater physical symptoms, mental strain, and fatigue with NSM than SSM [29].

### Mastectomy flap necrosis

The panel strongly considered the location of the incision to be a risk factor for severe mastectomy flap necrosis requiring re-operation (Fig. 2 and Supplementary Appendix 5). This is supported by results of a single-center retrospective review of 500 NSM procedures that showed a dramatic increase in the risk of mastectomy flap necrosis by the use of peri-areolar incisions, while the inframammary approach was associated with a decreased risk [30]. Another retrospective single-center study could not confirm this relationship [31].

The panel did not reach consensus or even a majority agreement when asked if the risk of flap necrosis depends on the surgical technique used for mastectomy flap dissection. This disagreement is mirrored by discussions in the literature. A prospective observational study at Memorial Sloan-Kettering Cancer Center assessed risk factors for skin necrosis in patients undergoing uni- or bi-lateral mastectomy and reconstruction. They found that the use of sharp dissection versus cautery significantly increased the odds of any skin flap necrosis in multivariable analysis (odds ratio 5.94, 95% CI 2.16–16.34, *p* < 0.001) [32]. Another single-center study did not confirm this association [31]. By contrast, the panel reached consensus that the amount and duration of pressure applied by retractors during surgery play an important role.

The panel considered skin flap thickness to be associated with risk of skin necrosis, which is supported by the limited published evidence [33]. In this single-center retrospective review, 10 NSMs with ischemic complications had significantly thinner NSM flaps as measured by post-operative magnetic resonance imaging (MRI) compared with 50 NSMs without ischemic complications (7.3 mm vs. 9.0 mm, *p* = 0.0280).

The panel was divided on the potential benefit of intra-operative skin flap viability assessment: almost half abstained, suggesting it is rarely used clinically and the other half was divided on its clinical value. One single-center study from Japan showed a dramatic decrease in the rate of full-thickness skin necrosis by the use of indocyanine green angiography-guided skin trimming from 17.8 to 4.8% (*p* < 0.05) [34]. During the iterative question review-and-adjust process preceding the conference, a plastic surgeon added a question addressing the concept of tissue pre-conditioning, for example by the local application of nitroglycerine. More than half of the panel abstained from a vote about the value of nitroglycerine, suggesting little experience in its use and very few were in favor. However, a randomized controlled trial (RCT) performed at the University of British Columbia was stopped for early success after 165 patients showed a difference in the rate of mastectomy flap necrosis of 18.5 percent in favor of nitroglycerine (*p* = 0.006; 95% CI 5.3–31.0%) [35]. The panel did not feel that early use of compression dressings or bras has any influence on the development or the prevention of flap necrosis. There was strong consensus that the risk of flap necrosis depends on the expertise and experience of the surgeon. The organization and promotion of high-quality surgical training is one of the key missions of the OPBC.

### Optimal thickness of mastectomy skin flap

There was a strong consensus that the thickness of the skin flaps should be determined by the place and depth of the superficial fascia and that dissection should follow this plane (Fig. 2 and Supplementary Appendix 5). This is in line with the conclusion of a recent literature review [36]. It showed that the variable thickness of the subcutis precludes adoption of a single specific universal thickness for mastectomy skin flaps.

### Inframammary fold incision

The panel did not recommend the use of an inframammary fold incision independent of breast size and shape, and was equally divided when asked if it can be used for tumors < 1 cm from the nipple on imaging (Fig. 2 and Supplementary Appendix 5). The inframammary fold incision seems to be the most popular and commonly used approach today [37, 38]. A retrospective single-center study demonstrated that refinements of surgical techniques, such as use of the inframammary fold incisions, can dramatically lower the rate of complications after NSM [39]. New and less invasive approaches are currently being evaluated. For example, a single-axillary-incision endoscopic hybrid technique was safe and associated with low morbidity and high patient satisfaction in an early patient series from Taiwan [40]. This hidden incision type can also be used for robotic assisted techniques that have the potential to further improve patient satisfaction although these techniques are not widely practiced at present [41, 42].

A bare majority felt that the inframammary approach should not be used for tumors in the upper inner quadrants in large breasts to avoid compromising oncological safety due to limited access that may increase the risk of positive margins. A retrospective analysis of a prospectively maintained single-center database showed that the majority of relapses (12 of 14; 85.7%) developed in the subcutaneous tissue of the quadrant where the primary tumor was located [43]. A large majority of the panel was of the opinion that the inframammary incision can be used independent of tumor size and location or breast size as long as the whole breast tissue is safely reached and removed and operative visibility is maintained. Finally, a majority of the panel discouraged a separate periareolar incision for retro-areolar frozen section to exclude nipple involvement before the inframammary fold is incised.

### Oncological contraindications to preservation of the skin envelope

A slim majority voted that cT4b and cT4c breast cancers limited to a 1 × 1 cm area of skin ulceration should be a contra-indication to skin preservation (Fig. 3 and Supplementary Appendix 6). However, other investigators have suggested that careful assessment of pathology and treatment response may identify clinical T4 patients appropriate for conservation of the skin envelope [44]. A clear majority of the panel felt that edema of the skin with enhancement on imaging should be a contraindication even without redness. The panel reached consensus that inflammatory breast cancer is an absolute contraindication even with complete remission of all skin changes after NACT. Half of the panel believed that the absence of any distance between tumor and skin on pre-operative imaging should be a contraindication, even in the absence of clinical signs of skin infiltration. A slim majority of panelists felt that a histological margin of less than 1 mm is no contraindication for the preservation of the skin flap, while 37% of panelists considered it a contraindication. During the discussion, it became obvious that there are wide international variations in clinical practice concerning positive or close margins after NSM, ranging from further surgery to post-mastectomy radiotherapy to no treatment. A large patient series at Mayo Clinic showed that the overall 5-year risk of local recurrence was higher in patients with a margin ≤ 2 mm compared to a margin > 2 mm (11.2% vs. 3.1%), with the proximity of the final margin being an independent significant risk factor for local recurrence [45]. However, in a retrospective single-center study of 64 SSM procedures with a positive mastectomy margin towards the skin in the area of the primary tumor, only 13 (20%) had residual cancer in simultaneous re-excisions of the subcutaneous tissue, suggesting a high incidence of false-positive margins [16].

### Oncological contraindications to preservation of the nipple

There was strong consensus that clinical signs of nipple involvement and any R1 resection at the nipple margin are contra-indications to nipple preservation (Fig. 3 and Supplementary Appendix 6). When the panel was asked if the nipple can be excised without the areola in patients with positive retroareolar margins, no consensus was reached. A majority felt that radiotherapy without excision should not be offered. Finally, no consensus was achieved concerning atypia in the margin on frozen section that is not 100% consistent with the diagnosis of DCIS or invasive cancer.

Even though the clinical relevance of a positive retroareolar margin is not known in the individual patient, the involvement of the NAC has traditionally been considered the main contraindication to NSM [46]. Local recurrence in the nipple can occur as a rare and late event after NSM. In a retrospective analysis of 861 patients, seven nipple recurrences were diagnosed on average 32 months after surgery [47]. In clinical practice, there are many ways to address a positive nipple margin: More surgery, radiotherapy (± boost), or observation [48]. A retrospective analysis of 217 mastectomy patients revealed that despite a high frequency of malignant nipple involvement in 10.6%, less than 1% had involvement of the areola [49]. A center that routinely performed further surgery in 46 patients with positive nipple margins showed that surgical practice differed considerably, with 51% of patients having only the nipple and 49% having the entire NAC excised [50].

The panel was generous in offering to attempt nipple preservation to patients with an increased risk of occult nipple involvement before surgery, such as patients with a tumor-nipple distance ≤ 1 cm or tumors > 3 cm in diameter. Tumor size, tumor-nipple distance, extensive DCIS component and multicentricity are consistent predictors of nipple involvement [51, 52]. Several studies have investigated the role of imaging, particularly MRI and ultrasound, in predicting the risk of nipple involvement, and suggest a minimum distance of 1–2 cm [53–55]. However, patient selection based on clinicopathologic characteristics is controversial, since a negative retroareolar margin may exclude occult nipple involvement with a high negative predictive value even in patients at high risk [51]. This is in support of the panel recommendations to treat the nipple margin like any other margin.

While a slim majority of the panel agreed that bloody nipple discharge is considered an oncological contraindication to nipple preservation, a clear majority felt that this does not apply to clear nipple discharge, since the presence of nipple discharge is not equivalent to NAC involvement [56].

### Risk of nipple necrosis

There was a consensus recommendation that skin reduction techniques with NAC pedicles or free nipple grafting can be offered to women with large and ptotic breasts as part of NSM irrespective of the type of reconstruction. A strong majority felt that a delayed procedure after pre-shaping the breast by reduction mammoplasty is a good approach. Indeed, several techniques have been described to reduce large and ptotic breasts during NSM, and the concept of delayed NSM after reduction mammoplasty is well supported by the literature as well [57–59].

### Special considerations in the risk reducing and therapeutic setting

The panel did not routinely recommend sentinel lymph node biopsy (SLNB) during risk reducing mastectomy, in line with a recent review of this topic (Fig. 4 and Supplementary Appendix 7) [60]. A majority felt that a pre-operative MRI without abnormal finding is a clear reason to omit SLNB. There was consensus not to remove the inframammary fold and the soft tissue all the way to the clavicle in both the risk reducing and therapeutic setting; rather, the breast tissue should be removed according to its individual anatomical borders. However, several panelists cautioned that personalizing the extent of surgery may have an impact on the effectiveness of the procedure. While the panel reached consensus that the fascia of the pectoral major muscle should not be removed during risk reducing NSM, only half of the panel felt that this also applies to therapeutic NSM. Similarly, while there was consensus against routine retroareolar frozen section in the risk reducing setting, only a bare majority felt that this recommendation also applies to the therapeutic setting.

### Preferred method of breast reconstruction

While a slim majority of panelists did not use synthetic meshes, the panel was divided when asked if they use acellular dermal matrix (ADM; Fig. 5 and Supplementary Appendix 8). The preferred method of reconstruction after NSM in a patient with small breasts and no planned radiotherapy varied widely. In fact, of eight different techniques, ranging from autologous to implant-based reconstruction with different timings and positioning of the implants, every option was chosen by at least one panelist. Interestingly, one-third of the panel chose immediate one-stage sub-pectoral implant-based reconstruction without synthetic mesh or ADM. The extensive literature on breast reconstruction after NSM has been summarized in Supplementary Appendix 3. The wide variation in clinical reconstruction practice that is mirrored in the literature calls for RCTs to guide treatment.

### Prospective registries

There was a clear consensus that patients undergoing NSM should be included in national and/or international prospective registries (Fig. 6 and Supplementary Appendix 9). One such registry that has been recommended during the discussion is the international NSM registry INSPIRE [61]. Indeed, a recent review of 11 observational cohort studies evaluating 7018 NSM, SSM and traditional mastectomy procedures concluded that these studies were of low quality [17]. Hence, there is a clear need for high-quality multi-center prospective studies to assess the efficacy and safety of NSM [62].

### Outcome assessment

The panel was unanimous in recommending the use of pre- and post-operative pictures as standard tool for objective outcome assessment after NSM (Fig. 6 and Supplementary Appendix 9), thereby recognizing the extent of such a commitment at high-volume centers. Since the association between objective esthetic outcomes and QoL is complex, the panel also endorsed the routine evaluation of PROs. The European Organisation for Research and Treatment of Cancer QLQ-BRECON23 questionnaire was proposed by one of the panelists as an internationally validated tool for standardized outcome assessment in patients undergoing breast reconstruction [63]. Almost half of the panelists abstained (presumably, because this is a new and not yet well-known questionnaire) and the other half was divided when asked if this tool should be used for outcome assessment. When asked about the well-established BREAST-Q reconstruction module, a majority of the panel recommended not using all scales for feasibility reasons due to its size, but a large majority voted for selected scales of the questionnaire as a standard tool for outcome assessment [10, 22, 64, 65].

## Conclusions

The OPBC panel acknowledged the need for standardization of various aspects of NSM and immediate breast reconstruction. It considers the procedure safe as long as specialists, who select the right patients and the appropriate techniques, perform it. The panel endorsed several oncological contraindications to the preservation of the skin and nipple. It recommended inclusion of patients in prospective registries and evaluation of PROs as part of routine outcome assessment in clinical practice and research. The consensus conference revealed considerable heterogeneity in breast reconstruction practice, which is mirrored in the current literature. This situation calls for RCTs to evaluate the safest and most efficacious reconstruction techniques.

## Electronic supplementary material

Below is the link to the electronic supplementary material.

Supplementary material 1 (DOCX 79 KB)

## References

[1] Cense HA, Rutgers EJ, Cardozo ML, Van Lanschot JJ (2001). Nipple-sparing mastectomy in breast cancer: a viable option?. Eur J Surg Oncol.

[2] Peled AW, Wang F, Foster RD (2016). Expanding the indications for total skin-sparing mastectomy: is it safe for patients with locally advanced disease?. Ann Surg Oncol.

[3] Sisco M, Kyrillos AM, Lapin BR, Wang CE, Yao KA (2016). Trends and variation in the use of nipple-sparing mastectomy for breast cancer in the United States. Breast Cancer Res Treat.

[4] Jakub JW, Peled AW, Gray RJ (2018). Oncologic safety of prophylactic nipple-sparing mastectomy in a population with BRCA mutations: a multi-institutional study. JAMA Surg.

[5] Yao K, Liederbach E, Tang R (2015). Nipple-sparing mastectomy in BRCA1/2 mutation carriers: an interim analysis and review of the literature. Ann Surg Oncol.

[6] Burdge EC, Yuen J, Hardee M (2013). Nipple skin-sparing mastectomy is feasible for advanced disease. Ann Surg Oncol.

[7] Sherman KA, Woon S, French J, Elder E (2017). Body image and psychological distress in nipple-sparing mastectomy: the roles of self-compassion and appearance investment. Psychooncology.

[8] Jagsi R, Li Y, Morrow M (2015). Patient-reported quality of life and satisfaction with cosmetic outcomes after breast conservation and mastectomy with and without reconstruction: results of a survey of breast cancer survivors. Ann Surg.

[9] Atisha DM, Rushing CN, Samsa GP (2015). A national snapshot of satisfaction with breast cancer procedures. Ann Surg Oncol.

[10] Bailey CR, Ogbuagu O, Baltodano PA (2017). Quality-of-life outcomes improve with nipple-sparing mastectomy and breast reconstruction. Plast Reconstr Surg.

[11] Metcalfe KA, Cil TD, Semple JL (2015). Long-term psychosocial functioning in women with bilateral prophylactic mastectomy: does preservation of the nipple-areolar complex make a difference?. Ann Surg Oncol.

[12] Oncoplastic Breast Consortium (2018) https://oncoplasticbc.org/. Accessed 20 Apr 2018

[13] Weber WP, Soysal SD, El-Tamer M (2017). First international consensus conference on standardization of oncoplastic breast conserving surgery. Breast Cancer Res Treat.

[14] Weber WP (2018) Oncoplastic Breast Consortium. https://oncoplasticbc.org/documents/protocol_mar_15_2018_opbc_cons_conf_nsm_20180305.pdf. Accessed 20 Apr 2018

[15] Lawrence R (1989) U. S. Preventive Services Task Force Edition. Guide to Clinical Preventive Services DIANE Publishing

[16] Cao D, Tsangaris TN, Kouprina N (2008). The superficial margin of the skin-sparing mastectomy for breast carcinoma: factors predicting involvement and efficacy of additional margin sampling. Ann Surg Oncol.

[17] Mota BS, Riera R, Ricci MD (2016). Nipple- and areola-sparing mastectomy for the treatment of breast cancer. Cochrane Database Syst Rev.

[18] Benediktsson KP, Perbeck L (2008). Survival in breast cancer after nipple-sparing subcutaneous mastectomy and immediate reconstruction with implants: a prospective trial with 13years median follow-up in 216 patients. Eur J Surg Oncol.

[19] Li M, Chen K, Liu F, Su F, Li S, Zhu L (2017). Nipple sparing mastectomy in breast cancer patients and long-term survival outcomes: an analysis of the SEER database. PLoS ONE.

[20] Gennari R, Griguolo G, Dieci MV (2016). Fat grafting for breast cancer patients: from basic science to clinical studies. Eur J Surg Oncol.

[21] Serra-Renom JM, Munoz-Olmo JL, Serra-Mestre JM (2010). Fat grafting in postmastectomy breast reconstruction with expanders and prostheses in patients who have received radiotherapy: formation of new subcutaneous tissue. Plast Reconstr Surg.

[22] Qureshi AA, Odom EB, Parikh RP, Myckatyn TM, Tenenbaum MM (2017). Patient-reported outcomes of aesthetics and satisfaction in immediate breast reconstruction after nipple-sparing mastectomy with implants and fat grafting. Aesthet Surg J.

[23] Bertolini F, Petit JY, Kolonin MG (2015). Stem cells from adipose tissue and breast cancer: hype, risks and hope. Br J Cancer.

[24] Masia J, Bordoni D, Pons G, Liuzza C, Castagnetti F, Falco G (2015). Oncological safety of breast cancer patients undergoing free-flap reconstruction and lipofilling. Eur J Surg Oncol.

[25] Myckatyn TM, Wagner IJ, Mehrara BJ (2017). Cancer risk after fat transfer: a multicenter case-cohort study. Plast Reconstr Surg.

[26] Waked K, Colle J, Doornaert M, Cocquyt V, Blondeel P (2017). Systematic review: the oncological safety of adipose fat transfer after breast cancer surgery. Breast.

[27] De Decker M, De Schrijver L, Thiessen F, Tondu T, Van Goethem M, Tjalma WA (2016). Breast cancer and fat grafting: efficacy, safety and complications—a systematic review. Eur J Obstet Gynecol Reprod Biol.

[28] Manning AT, Wood C, Eaton A (2015). Nipple-sparing mastectomy in patients with BRCA1/2 mutations and variants of uncertain significance. Br J Surg.

[29] Jackson RS, Sanders T, Park A (2017). Prospective study comparing surgeons’ pain and fatigue associated with nipple-sparing versus skin-sparing mastectomy. Ann Surg Oncol.

[30] Colwell AS, Tessler O, Lin AM (2014). Breast reconstruction following nipple-sparing mastectomy: predictors of complications, reconstruction outcomes, and 5year trends. Plast Reconstr Surg.

[31] Donovan CA, Harit AP, Chung A, Bao J, Giuliano AE, Amersi F (2016). Oncological and surgical outcomes after nipple-sparing mastectomy: do incisions matter?. Ann Surg Oncol.

[32] Matsen CB, Mehrara B, Eaton A (2016). Skin flap necrosis after mastectomy with reconstruction: a prospective study. Ann Surg Oncol.

[33] Frey JD, Salibian AA, Choi M, Karp NS (2017). Mastectomy flap thickness and complications in nipple-sparing mastectomy: objective evaluation using magnetic resonance imaging. Plast Reconstr Surg Glob Open.

[34] Gorai K, Inoue K, Saegusa N (2017). Prediction of skin necrosis after mastectomy for breast cancer using indocyanine green angiography imaging. Plast Reconstr Surg Glob Open.

[35] Gdalevitch P, Van Laeken N, Bahng S (2015). Effects of nitroglycerin ointment on mastectomy flap necrosis in immediate breast reconstruction: a randomized controlled trial. Plast Reconstr Surg.

[36] Robertson SA, Rusby JE, Cutress RI (2014). Determinants of optimal mastectomy skin flap thickness. Br J Surg.

[37] Colwell AS, Christensen JM (2017). Nipple-sparing mastectomy and direct-to-implant breast reconstruction. Plast Reconstr Surg.

[38] Salibian AH, Harness JK, Mowlds DS (2013). Inframammary approach to nipple-areola-sparing mastectomy. Plast Reconstr Surg.

[39] Garwood ER, Moore D, Ewing C (2009). Total skin-sparing mastectomy: complications and local recurrence rates in 2 cohorts of patients. Ann Surg.

[40] Lai HW, Lin SL, Chen ST (2018). Single-axillary-incision endoscopic-assisted hybrid technique for nipple-sparing mastectomy: technique, preliminary results, and patient-reported cosmetic outcome from preliminary 50 procedures. Ann Surg Oncol.

[41] Sarfati B, Honart JF, Leymarie N, Rimareix F, Al Khashnam H, Kolb F (2017). Robotic da Vinci Xi-assisted nipple-sparing mastectomy: first clinical report. Breast J.

[42] Toesca A, Peradze N, Galimberti V (2017). Robotic nipple-sparing mastectomy and immediate breast reconstruction with implant: first report of surgical technique. Ann Surg.

[43] Cont NT, Maggiorotto F, Martincich L (2017). Primary tumor location predicts the site of local relapse after nipple–areola complex (NAC) sparing mastectomy. Breast Cancer Res Treat.

[44] Murphy BL, Hoskin TL, Boughey JC (2016). Contemporary operative management of T4 breast cancer. Surgery.

[45] Glorioso JM, Gonzalez Juarrero AB, Rodysill BR (2017). Margin proximity correlates with local recurrence after mastectomy for patients not receiving adjuvant radiotherapy. Ann Surg Oncol.

[46] Veronesi U, Stafyla V, Petit JY, Veronesi P (2012). Conservative mastectomy: extending the idea of breast conservation. Lancet Oncol.

[47] Lohsiriwat V, Martella S, Rietjens M (2012). Paget’s disease as a local recurrence after nipple-sparing mastectomy: clinical presentation, treatment, outcome, and risk factor analysis. Ann Surg Oncol.

[48] Amara D, Peled AW, Wang F, Ewing CA, Alvarado M, Esserman LJ (2015). Tumor Involvement of the nipple in total skin-sparing mastectomy: strategies for management. Ann Surg Oncol.

[49] Skousen J, Simmons J, McDonald LM, Ziemkiewicz P (2002). Acid-base accounting to predict post-mining drainage quality on surface mines. J Environ Qual.

[50] Tang R, Coopey SB, Merrill AL (2016). Positive nipple margins in nipple-sparing mastectomies: rates, management, and oncologic safety. J Am Coll Surg.

[51] Brachtel EF, Rusby JE, Michaelson JS (2009). Occult nipple involvement in breast cancer: clinicopathologic findings in 316 consecutive mastectomy specimens. J Clin Oncol.

[52] Zhang H, Li Y, Moran MS, Haffty BG, Yang Q (2015). Predictive factors of nipple involvement in breast cancer: a systematic review and meta-analysis. Breast Cancer Res Treat.

[53] Dent BL, Miller JA, Eden DJ, Swistel A, Talmor M (2017). Tumor-to-nipple distance as a predictor of nipple involvement: expanding the inclusion criteria for nipple-sparing mastectomy. Plast Reconstr Surg.

[54] Steen ST, Chung AP, Han SH, Vinstein AL, Yoon JL, Giuliano AE (2013). Predicting nipple-areolar involvement using preoperative breast MRI and primary tumor characteristics. Ann Surg Oncol.

[55] D’Alonzo M, Martincich L, Biglia N (2012). Clinical and radiological predictors of nipple-areola complex involvement in breast cancer patients. Eur J Cancer.

[56] Chang RY, Cheung PS (2017). Nipple preservation in breast cancer associated with nipple discharge. World J Surg.

[57] Salibian AH, Harness JK, Mowlds DS (2016). Primary buttonhole mastopexy and nipple-sparing mastectomy: a preliminary report. Ann Plast Surg.

[58] Gunnarsson GL, Bille C, Reitsma LC, Wamberg P, Thomsen JB (2017). Prophylactic nipple-sparing mastectomy and direct-to-implant reconstruction of the large and ptotic breast: is preshaping of the challenging breast a key to success?. Plast Reconstr Surg.

[59] Alperovich M, Tanna N, Samra F (2013). Nipple-sparing mastectomy in patients with a history of reduction mammaplasty or mastopexy: how safe is it?. Plast Reconstr Surg.

[60] Nagaraja V, Edirimanne S, Eslick GD (2016). Is sentinel lymph node biopsy necessary in patients undergoing prophylactic mastectomy? A systematic review and meta-analysis. Breast J.

[61] ESSO European Society of Surgical Oncology (2018) https://www.essoweb.org/eurecca-inspire. Accessed 20 Apr 2018

[62] De La Cruz L, Moody AM, Tappy EE, Blankenship SA, Hecht EM (2015). Overall survival, disease-free survival, local recurrence, and nipple-areolar recurrence in the setting of nipple-sparing mastectomy: a meta-analysis and systematic review. Ann Surg Oncol.

[63] Winters ZE, Afzal M, Rutherford C (2018). International validation of the European Organisation for research and treatment of cancer QLQ-BRECON23 quality-of-life questionnaire for women undergoing breast reconstruction. Br J Surg.

[64] Jagsi R, Momoh AO, Qi J (2018). Impact of radiotherapy on complications and patient-reported outcomes after breast reconstruction. J Natl Cancer Inst.

[65] Wei CH, Scott AM, Price AN (2016). Psychosocial and sexual well-being following nipple-sparing mastectomy and reconstruction. Breast J.

