# Supplementary material for: Oncoplastic Breast Consortium consensus conference on nipple-sparing mastectomy
Source: Breast Cancer Res Treat. 2018 Sep 4;172(3):523–37. doi: 10.1007/s10549-018-4937-1 (PMC6245050; doi:10.1007/s10549-018-4937-1)
Supplement: Supplementary file 1 — Supplementary material 1 (DOCX 79 KB) [file 10549_2018_4937_MOESM1_ESM.docx]

**Supplementary appendix 1. Oncoplastic Breast Consortium panel for consensus conference on nipple-sparing mastectomy**

| **Country** | **Number of panelists** | **Number of surgeons** | **Number of gynecologists** | **Number of plastic surgeons** | **Present at meeting in Basel** |
| --- | --- | --- | --- | --- | --- |
| Austria | 5 | 2 | 2 | 1 | 4 |
| Brazil | 4 | 0 | 4 | 0 | 4 |
| Germany | 3 | 0 | 3 | 0 | 3 |
| Hungary | 1 | 1 | 0 | 0 | 0 |
| Italy | 3 | 3 | 0 | 0 | 2 |
| Ireland | 1 | 1 | 0 | 0 | 1 |
| Israel | 3 | 3 | 0 | 0 | 3 |
| Portugal | 2 | 2 | 0 | 0 | 1 |
| Spain | 1 | 1 | 0 | 0 | 1 |
| Sweden | 2 | 2 | 0 | 0 | 2 |
| Switzerland | 11 | 3 | 6 | 2 | 11 |
| Thailand | 1 | 0 | 0 | 1 | 1 |
| UK | 4 | 4 | 0 | 0 | 4 |
| USA | 3 | 2 | 0 | 1 | 1 |
| **Total** | 44 | 24 | 15 | 5 | 38 |

The panel was also attended by a patient advocate who is not included in the above list.

**Supplementary appendix 2. Selected nipple sparing mastectomy series (2000–2018)**

| **Author, year** | **Study period** | **No. patients / No. procedures** | **Age in years (range)** | **Prophylactic setting  n (% of all patients)** | **Stage: AJCC or TNM** | **Follow-up in months** | **Local**  **recurrence**  **n (%)** | **Regional recurrence**  **n (%)** | | **NAC**  **recurrence**  **n (%)** | **Distant recurrence**  **n (%)** |
| --- | --- | --- | --- | --- | --- | --- | --- | --- | --- | --- | --- |
| Caruso^1^, 2006 | 1994–2004 | 50/NR | 42 (28–68) | 0 | 0-III | 66 | 0 | 0 | | 1 | 5 (10) |
| Benediktsson^2^, 2007 | 1988–1994 | 216/NR | 52.8 (29–81) | 0 | 0-III | 156 | 52 (24) | 4 (1.9) | | NR | 44 (20.3) |
| Voltura^3^, 2008 | 2002–2007 | 36/51 | 48 (32–75) | 5 (13.8) | 0-III | 18 | 2 (5.9) | 0 | | 0 | 0 |
| Crowe^4^, 2008 | 2001–2007 | 110/149 | 43 (20–72) | 27 (25) | 0-NR | 41 | 1 (0.9) | 1 (0.9) | | 0 | 2 (1.8) |
| Sakamoto^5^, 2009 | 2002–2005 | 87/NR | 50 | 0 | 0-IIIA | 52 | 0 | 0 | | 0 | 9 (10.3) |
| Gerber^6^, 2009 | 1994–2000 | 60/60 | 46 (45.1–50.9) | 0 | 0-IIIB | 101 | 5 (11.7) | 1 (1.7) | | 1 (1.7) | 14 (23.3) |
| Paepke^7^, 2009 | 2003–2006 | 96/109 | NA | 15 (16) | 0-IIIA | 34 | 1 (1) | 1 (1) | | 0 | 2 (2) |
| Harness^8^, 2011 | 2004–2009 | 43/60 | 48.7 (28–76) | NR | 0-IV | 18.5 | 0 | 0 | | 1 (2.3) | NR |
| Boneti^9^, 2011 | 1998–2010 | 141/227 | 53.1 | NR | NR | NR | — 7 (5) — | |  | NR | NR |
| de Alcantara^10^, 2011 | 2000–2010 | 200/353 | 44 (23–69) | 79 (39.5) | 0-III | NR | 0 | 0 | | 0 | 1 (0.5) |
| Jensen^11^, 2011 | 1997–2008 | 99/149 | 51 | 0 | I-III | 60.2 | 3 (3) | 0 | | 0 | 1 (1) |
| Spear^12^, 2011 | 1989–2010 | 101/161 | 44 (26–68) | 80 (79) | 0-pT2 | 43 | 0 | 0 | | 0 | NR |
| Loshiriwat^13^, 2012 | 2002–2008 | NR/861 | NA | 0 | pTis-pT3 | 50 | 36 (4.18) | 0 | | 7 (0.8) | 0 |
| Peled^14^, 2012 | 2001–2010 | 428/657 | 46.9 (19–78.3) | NR | 0-IV | 28 | — 4 (1) — | |  | 0 | 8 (1.9) |
| Petit^15^, 2012 | 2002–2007 | 934 /934 | NA | 0 | I-IIIA | 50 | 28 (3.6) invasive group / 9 (4.9) in situ group | |  | 11 (0.8) | NR |
| Coopey^16^, 2013 | 2007–2012 | 370/645 | 47 (23–78) | NR | pT0-pT3 | 22 | NR | NR | | NR | NR |
| Burdge^17^, 2013 | 2001–2013 | 39/39 | 50 (39.4–61) | 0 | II-IIIA | 18 | 5 (12.8) | 0 | | 0 | 1 (1.7) |
| Adam^18^, 2014 | 2000–2012 | 67/69 | NA | 0 | pT1-pT4 | 36 | 0 | 0 | | 0 | 0 |
| Yao^19^, 2015 | 2007–2014 | 201/397 | 41.3 (25–74) | 150 (74.6) | 0-III | 32.6 | 1 (0.5) | 3 (1.5) | | 0 | 1 (0.5) |
| Manning^20^, 2015 | 2005–2013 | 89/177 | NA | 63 (71) | 0-II | 28 | 0 | 0 | | 0 | NR |
| Krajewski^21^, 2015 | 2009–2014 | 341/566 | 48 (21–75) | NR | 0-≥II | 19 | 2 (0.6) | 3 (0.9) | | 0 | 7 (2) |
| Santoro^22^, 2015 | 2007–2015 | 186/186 | 44 | 0 | I-III | 35 | 3 (1.6) | NR | | 0 | 16 (8.6) |
| Peled^23^, 2016 | 2005–2013 | 139/NR | 46.9 | NR | IIb-III | 41 | — 10 (7.2) — | |  | 0 | 24 (17.2) |
| Moo^24^, 2016 | 2007–2013 | 413/721 | 48 (25–68) | 45 (10.9) | 0-III | 36 | 7 (10) | 1 (2) | | 1 (2) | 9 (2.4) |
| Tang^25^, 2016 | 2007–2014 | 766/1326 | 47 (21–78) | NR | NR | 36 | 9 (0.7) | 0 | | 0 | NR |
| Kurian^26^, 2016 | 1988–2013 | 993/993 | NA | NR | 0-III | 22.8 | NR | NR | | NR | NR |
| Orzalesi^27^, 2016 | 2009–2014 | 913/1006 | 47 (21–77) | 120 (12) | 0-III | 36.2 | — 22 (2.9) — | |  | 7 (1.0) | 5 (0.7) |
| Chan^28^, 2017 | 2009–2015 | 91/103 | 44.7 (27–68) | 3 (3.3) | 0-IV | 20.6 | 1 (1) | 1 (1) | | 1 (1) | 2 (2) |
| Smith^29^, 2017 | 2007–2016 | 297/311 | 48 (28–78) | 0 | 0-III | 51 | 4 (1.3) | 7 (2.4) | | 0 | 8 (2.7) |
| Cont^30^, 2017 | 2010–2015 | 518/NR | NA | 0 | NR | 33 | 14 (2.7) | 0 | | 1 (0.3) | 0 |
| Jakub^31^, 2018 | 1968–2013 | 346/548 | 41 (34.5–47.5) | 346 (100) | 0 | 34 | 0 | 0 | | 0 | 0 |
| NR = not reported, NA = not applicable, AJCC = American Joint Committee on Cancer, TNM = TNM Classification of malignant tumors | | | | | | | | | | | |

**Bibliography for supplementary appendix 2.**

1. Caruso F, Ferrara M, Castiglione G, et al. Nipple sparing subcutaneous mastectomy: sixty-six months follow-up. *Eur J Surg Oncol.* 2006;32(9):937-940.

2. Benediktsson KP, Perbeck L. Survival in breast cancer after nipple-sparing subcutaneous mastectomy and immediate reconstruction with implants: a prospective trial with 13 years median follow-up in 216 patients. *Eur J Surg Oncol.* 2008;34(2):143-148.

3. Voltura AM, Tsangaris TN, Rosson GD, et al. Nipple-sparing mastectomy: critical assessment of 51 procedures and implications for selection criteria. *Ann Surg Oncol.* 2008;15(12):3396-3401.

4. Crowe JP, Patrick RJ, Yetman RJ, Djohan R. Nipple-sparing mastectomy update: one hundred forty-nine procedures and clinical outcomes. *Arch Surg.* 2008;143(11):1106-1110; discussion 1110.

5. Sakamoto N, Fukuma E, Higa K, et al. Early results of an endoscopic nipple-sparing mastectomy for breast cancer. *Ann Surg Oncol.* 2009;16(12):3406-3413.

6. Gerber B, Krause A, Dieterich M, Kundt G, Reimer T. The oncological safety of skin sparing mastectomy with conservation of the nipple-areola complex and autologous reconstruction: an extended follow-up study. *Ann Surg.* 2009;249(3):461-468.

7. Paepke S, Schmid R, Fleckner S, et al. Subcutaneous mastectomy with conservation of the nipple-areola skin: broadening the indications. *Ann Surg.* 2009;250(2):288-292.

8. Harness JK, Vetter TS, Salibian AH. Areola and nipple-areola-sparing mastectomy for breast cancer treatment and risk reduction: report of an initial experience in a community hospital setting. *Ann Surg Oncol.* 2011;18(4):917-922.

9. Boneti C, Yuen J, Santiago C, et al. Oncologic safety of nipple skin-sparing or total skin-sparing mastectomies with immediate reconstruction. *J Am Coll Surg.* 2011;212(4):686-693; discussion 693-685.

10. de Alcantara Filho P, Capko D, Barry JM, Morrow M, Pusic A, Sacchini VS. Nipple-sparing mastectomy for breast cancer and risk-reducing surgery: the Memorial Sloan-Kettering Cancer Center experience. *Ann Surg Oncol.* 2011;18(11):3117-3122.

11. Jensen JA, Orringer JS, Giuliano AE. Nipple-sparing mastectomy in 99 patients with a mean follow-up of 5 years. *Ann Surg Oncol.* 2011;18(6):1665-1670.

12. Spear SL, Willey SC, Feldman ED, et al. Nipple-sparing mastectomy for prophylactic and therapeutic indications. *Plast Reconstr Surg.* 2011;128(5):1005-1014.

13. Lohsiriwat V, Martella S, Rietjens M, et al. Paget's disease as a local recurrence after nipple-sparing mastectomy: clinical presentation, treatment, outcome, and risk factor analysis. *Ann Surg Oncol.* 2012;19(6):1850-1855.

14. Warren Peled A, Foster RD, Stover AC, et al. Outcomes after total skin-sparing mastectomy and immediate reconstruction in 657 breasts. *Ann Surg Oncol.* 2012;19(11):3402-3409.

15. Petit JY, Veronesi U, Orecchia R, et al. Risk factors associated with recurrence after nipple-sparing mastectomy for invasive and intraepithelial neoplasia. *Ann Oncol.* 2012;23(8):2053-2058.

16. Coopey SB, Tang R, Lei L, et al. Increasing eligibility for nipple-sparing mastectomy. *Ann Surg Oncol.* 2013;20(10):3218-3222.

17. Burdge EC, Yuen J, Hardee M, et al. Nipple skin-sparing mastectomy is feasible for advanced disease. *Ann Surg Oncol.* 2013;20(10):3294-3302.

18. Adam H, Bygdeson M, de Boniface J. The oncological safety of nipple-sparing mastectomy - a Swedish matched cohort study. *Eur J Surg Oncol.* 2014;40(10):1209-1215.

19. Yao K, Liederbach E, Tang R, et al. Nipple-sparing mastectomy in BRCA1/2 mutation carriers: an interim analysis and review of the literature. *Ann Surg Oncol.* 2015;22(2):370-376.

20. Manning AT, Wood C, Eaton A, et al. Nipple-sparing mastectomy in patients with BRCA1/2 mutations and variants of uncertain significance. *Br J Surg.* 2015;102(11):1354-1359.

21. Krajewski AC, Boughey JC, Degnim AC, et al. Expanded Indications and Improved Outcomes for Nipple-Sparing Mastectomy Over Time. *Ann Surg Oncol.* 2015;22(10):3317-3323.

22. Santoro S, Loreti A, Cavaliere F, et al. Neoadjuvant chemotherapy is not a contraindication for nipple sparing mastectomy. *Breast.* 2015;24(5):661-666.

23. Peled AW, Wang F, Foster RD, et al. Expanding the Indications for Total Skin-Sparing Mastectomy: Is It Safe for Patients with Locally Advanced Disease? *Ann Surg Oncol.* 2016;23(1):87-91.

24. Moo TA, Pinchinat T, Mays S, et al. Oncologic Outcomes After Nipple-Sparing Mastectomy. *Ann Surg Oncol.* 2016;23(10):3221-3225.

25. Tang R, Coopey SB, Merrill AL, et al. Positive Nipple Margins in Nipple-Sparing Mastectomies: Rates, Management, and Oncologic Safety. *J Am Coll Surg.* 2016;222(6):1149-1155.

26. Kurian AW, Canchola AJ, Gomez SL, Clarke CA. Equivalent survival after nipple-sparing compared to non-nipple-sparing mastectomy: data from California, 1988-2013. *Breast Cancer Res Treat.* 2016;160(2):333-338.

27. Orzalesi L, Casella D, Santi C, et al. Nipple sparing mastectomy: Surgical and oncological outcomes from a national multicentric registry with 913 patients (1006 cases) over a six year period. *Breast.* 2016;25:75-81.

28. Chan YH, Yau WM, Cheung PS. Oncological Safety of Technical Feasibility of Nipple-Sparing Mastectomy for Breast Cancer: The Hong Kong Experience *World J Surg* 2018;42(5):1375-1383.

29. Smith BL, Tang R, Rai U, et al. Oncologic Safety of Nipple-Sparing Mastectomy in Women with Breast Cancer. *J Am Coll Surg.* 2017;225(3):361-365.

30. Cont NT, Maggiorotto F, Martincich L, et al. Primary tumor location predicts the site of local relapse after nipple-areola complex (NAC) sparing mastectomy. *Breast Cancer Res Treat.* 2017;165(1):85-95.

31. Jakub JW, Peled AW, Gray RJ, et al. Oncologic Safety of Prophylactic Nipple-Sparing Mastectomy in a Population With BRCA Mutations: A Multi-institutional Study. *JAMA Surg.* 2018;153(2):123-129.

**Supplementary appendix 3: Review of the literature on breast reconstruction after nipple-sparing mastectomy**

*Breast reconstruction without planned radiotherapy*Jagsi and colleagues using the MarketScan database found that the use of implants increased and the use of autologous techniques decreased over time from 1998 to 2007 in the United States (p < 0.001).^1^ Compared with implant-based reconstructions, all types of autologous reconstructions were associated with a higher risk of complications in a prospective multi-center cohort study in the United States and Canada, which has been shown to negatively impact quality of life (QoL) in another large prospective cohort in the United Kingdom.^2,3^ In contrast, unplanned surgical revision rates were highest after tissue expander-based compared with autologous reconstruction or direct-to-implant reconstruction in a retrospective cohort study of 15’154 women (tissue expander = 59.2% vs autologous 34.4% vs direct-to-implant 45.9%, p < 0.001).^4^ Importantly, two large surveys of breast cancer survivors in the United States showed the advantages of autologous reconstruction when assessing patient-reported outcomes (PROs) with significantly lower patient satisfaction scores after implant-based reconstruction.^5,6^

The traditional approach to implant-based reconstruction is the positioning of a tissue expander underneath the pectoral muscle, which is replaced by the final implant in a second procedure. The classical two-stage approach has been modified by leading experts with great success by positioning the expander in the subcutaneous plane in adequately selected patients.^7,8^ A newer concept is direct-to-implant, which can be done in the sub-pectoral or pre-pectoral pocket and is commonly augmented with acellular dermal matrix (ADM).^9^ PROs have been shown to be comparable between direct-to-implant and tissue expander/implant reconstructions.^10^

In a multi-center randomised controlled trial (RCT) from the Netherlands in 142 patients undergoing SSM, one-stage sub-pectoral implant-based breast reconstruction with ADM was compared with two-stage implant-based breast reconstruction. It was associated with significantly higher risk of surgical complications (crude odds ratio 3.81, 95% CI 2.67–5.43, p < 0.001), reoperation (3.38, 95% CI 2.10–5.45, p < 0.001), and removal of implant, ADM, or both (8.80, 95% CI 8.24–9.40, p < 0.001).^11^ However, others have used the sub-pectoral direct-to-implant reconstruction augmented with ADM with great success.^12,13^ The use of immediate direct-to-sub-pectoral-implants even without the use of ADM or synthetic mesh has recently been shown to be associated with low rates of complications in small series when focusing on patients with thick skin flaps.^14^ Concerning pre-pectoral positioning, early single-center or even single-surgeon series have experienced very short inpatient stays using full ADM coverage and complication rates that were comparable to the sub-pectoral positioning.^15,16^ Interestingly, even immediate single-stage reconstruction with pre-pectoral silicone implants without ADM or mesh is considered an acceptable and safe treatment option by some.^17^ A more inexpensive alternative to ADM is synthetic mesh, which has been used by several groups with acceptable complication rates and high QoL scores.^18-20^

*Breast reconstruction with planned radiotherapy*Radiotherapy is recognized as risk factor for complications and surgical revisions after NSM.^21^ In a large observational study of 5219 patients, radiotherapy significantly increased the risk of implant removal (odds ratio = 1.48; p < 0.001) and, after autologous reconstruction, fat necrosis (odds ratio = 1.55; p = 0.01).^22^ Nevertheless, several large series have shown that good outcomes can be achieved by implant-based reconstruction in the context of radiotherapy, with different timing strategies for the two-stage approach.^23,24^ However, a prospective multi-center cohort study compared complications and PROs of 622 irradiated and 1625 non-irradiated patients who received reconstruction between 2012 and 2015.^25^ Autologous reconstruction was associated with a lower risk of complications (OR = 0.47, 95% CI = 0.27 to 0.82, p = 0.007) and a higher BREAST-Q satisfaction with breasts score (63.5 vs 47.7; p=0.002). Two other studies confirmed these findings.^5,26^

**Bibliography for supplementary appendix 3:**

1. Jagsi R, Jiang J, Momoh AO, et al. Trends and variation in use of breast reconstruction in patients with breast cancer undergoing mastectomy in the United States. J Clin Oncol 2014; 32(9): 919-26.

2. Wilkins EG, Hamill JB, Kim HM, et al. Complications in Postmastectomy Breast Reconstruction: One-year Outcomes of the Mastectomy Reconstruction Outcomes Consortium (MROC) Study. Ann Surg 2018; 267(1): 164-70.

3. Browne JP, Jeevan R, Gulliver-Clarke C, Pereira J, Caddy CM, van der Meulen JHP. The association between complications and quality of life after mastectomy and breast reconstruction for breast cancer. Cancer 2017; 123(18): 3460-7.

4. Fischer JP, Fox JP, Nelson JA, Kovach SJ, Serletti JM. A Longitudinal Assessment of Outcomes and Healthcare Resource Utilization After Immediate Breast Reconstruction-Comparing Implant- and Autologous-based Breast Reconstruction. Ann Surg 2015; 262(4): 692-9.

5. Jagsi R, Li Y, Morrow M, et al. Patient-reported Quality of Life and Satisfaction With Cosmetic Outcomes After Breast Conservation and Mastectomy With and Without Reconstruction: Results of a Survey of Breast Cancer Survivors. Ann Surg 2015; 261(6): 1198-206.

6. Atisha DM, Rushing CN, Samsa GP, et al. A national snapshot of satisfaction with breast cancer procedures. Ann Surg Oncol 2015; 22(2): 361-9.

7. Bettinger LN, Waters LM, Reese SW, Kutner SE, Jacobs DI. Comparative Study of Prepectoral and Subpectoral Expander-Based Breast Reconstruction and Clavien IIIb Score Outcomes. Plast Reconstr Surg Glob Open 2017; 5(7): e1433.

8. Sbitany H, Piper M, Lentz R. Prepectoral Breast Reconstruction: A Safe Alternative to Submuscular Prosthetic Reconstruction following Nipple-Sparing Mastectomy. Plast Reconstr Surg 2017; 140(3): 432-43.

9. Colwell AS, Christensen JM. Nipple-Sparing Mastectomy and Direct-to-Implant Breast Reconstruction. Plast Reconstr Surg 2017; 140(5S Advances in Breast Reconstruction): 44S-50S.

10. Qureshi AA, Odom EB, Parikh RP, Myckatyn TM, Tenenbaum MM. Patient-Reported Outcomes of Aesthetics and Satisfaction in Immediate Breast Reconstruction After Nipple-Sparing Mastectomy With Implants and Fat Grafting. Aesthet Surg J 2017; 37(9): 999-1008.

11. Dikmans RE, Negenborn VL, Bouman MB, et al. Two-stage implant-based breast reconstruction compared with immediate one-stage implant-based breast reconstruction augmented with an acellular dermal matrix: an open-label, phase 4, multicentre, randomised, controlled trial. Lancet Oncol 2017; 18(2): 251-8.

12. Salzberg CA, Ashikari AY, Koch RM, Chabner-Thompson E. An 8-year experience of direct-to-implant immediate breast reconstruction using human acellular dermal matrix (AlloDerm). Plast Reconstr Surg 2011; 127(2): 514-24.

13. Choi M, Frey JD, Alperovich M, Levine JP, Karp NS. "Breast in a Day": Examining Single-Stage Immediate, Permanent Implant Reconstruction in Nipple-Sparing Mastectomy. Plast Reconstr Surg 2016; 138(2): 184e-91e.

14. Rancati AO, Angrigiani CH, Hammond DC, et al. Direct to Implant Reconstruction in Nipple Sparing Mastectomy: Patient Selection by Preoperative Digital Mammogram. Plast Reconstr Surg Glob Open 2017; 5(6): e1369.

15. Sigalove S, Maxwell GP, Sigalove NM, et al. Prepectoral Implant-Based Breast Reconstruction and Postmastectomy Radiotherapy: Short-Term Outcomes. Plast Reconstr Surg Glob Open 2017; 5(12): e1631.

16. Woo A, Harless C, Jacobson SR. Revisiting an Old Place: Single-Surgeon Experience on Post-Mastectomy Subcutaneous Implant-Based Breast Reconstruction. Breast J 2017; 23(5): 545-53.

17. Singla A, Singla A, Lai E, Caminer D. Subcutaneously Placed Breast Implants after a Skin-Sparing Mastectomy: Do We Always Need ADM? Plast Reconstr Surg Glob Open 2017; 5(7): e1371.

18. Dieterich M, Paepke S, Zwiefel K, et al. Implant-based breast reconstruction using a titanium-coated polypropylene mesh (TiLOOP Bra): a multicenter study of 231 cases. Plast Reconstr Surg 2013; 132(1): 8e-19e.

19. Casella D, Di Taranto G, Marcasciano M, et al. Nipple-sparing bilateral prophylactic mastectomy and immediate reconstruction with TiLoop((R)) Bra mesh in BRCA1/2 mutation carriers: A prospective study of long-term and patient reported outcomes using the BREAST-Q. Breast 2018; 39: 8-13.

20. Pukancsik D, Kelemen P, Gulyas G, et al. Clinical experiences with the use of ULTRAPRO((R)) mesh in single-stage direct-to-implant immediate postmastectomy breast reconstruction in 102 patients: A retrospective cohort study. Eur J Surg Oncol 2017; 43(7): 1244-51.

21. Reish RG, Lin A, Phillips NA, et al. Breast reconstruction outcomes after nipple-sparing mastectomy and radiation therapy. Plast Reconstr Surg 2015; 135(4): 959-66.

22. Jagsi R, Jiang J, Momoh AO, et al. Complications After Mastectomy and Immediate Breast Reconstruction for Breast Cancer: A Claims-Based Analysis. Ann Surg 2016; 263(2): 219-27.

23. Cordeiro PG, Albornoz CR, McCormick B, et al. What Is the Optimum Timing of Postmastectomy Radiotherapy in Two-Stage Prosthetic Reconstruction: Radiation to the Tissue Expander or Permanent Implant? Plast Reconstr Surg 2015; 135(6): 1509-17.

24. Kronowitz SJ, Lam C, Terefe W, et al. A multidisciplinary protocol for planned skin-preserving delayed breast reconstruction for patients with locally advanced breast cancer requiring postmastectomy radiation therapy: 3-year follow-up. Plast Reconstr Surg 2011; 127(6): 2154-66.

25. Jagsi R, Momoh AO, Qi J, et al. Impact of Radiotherapy on Complications and Patient-Reported Outcomes After Breast Reconstruction. J Natl Cancer Inst 2018; 110(2).

26. Chawla AK, Kachnic LA, Taghian AG, Niemierko A, Zapton DT, Powell SN. Radiotherapy and breast reconstruction: complications and cosmesis with TRAM versus tissue expander/implant. Int J Radiat Oncol Biol Phys 2002; 54(2): 520-6.

**Supplementary appendix 4. Consensus conference results: Standardization, oncological safety and indications**

|  | | **No. of responses** | **Percentage votes** | | | | | | **Voting result*** | |
| --- | --- | --- | --- | --- | --- | --- | --- | --- | --- | --- |
|  |  |  | **Yes (n)** | | **No (n)** | | **Abstain (n)** | |  | |
| **In the context of NSM, there is a need for standardization of** | | | | | | | | | | |
| - | Indications | 42 | 83% | (35) | 17% | (7) | 0% | (0) | Yes | (Consensus) |
| - | Contraindications | 41 | 90% | (37) | 10% | (4) | 0% | (0) | Yes | (Consensus) |
| - | Surgical technique | 44 | 68% | (30) | 27% | (12) | 5% | (2) | Yes | (Majority) |
| - | Type and timing of reconstruction in the setting of  adjuvant radiotherapy | 44 | 61% | (27) | 27% | (12) | 11% | (5) | Yes | (Majority) |
| - | Outcome assessment | 43 | 91% | (39) | 7% | (3) | 2% | (1) | Yes | (Consensus) |
| - | Radiological follow-up | 44 | 64% | (28) | 27% | (12) | 9% | (4) | Yes | (Majority) |
|  | | | | | | | | | | |
| **Based on the limited level of evidence and your experience, the oncological safety of NSM is** | | | | | | | | | | |
| - | Comparable to conventional mastectomy  without immediate reconstruction | 43 | 65% | (28) | 26% | (11) | 9% | (4) | Yes | (Majority) |
| - | Comparable to skin sparing mastectomy | 43 | 86% | (37) | 7% | (3) | 7% | (3) | Yes | (Consensus) |
| - | Comparable to breast conserving therapy | 39 | 62% | (24) | 18% | (7) | 21% | (8) | Yes | (Majority) |
| - | Compromised by the use of immediate fat grafting | 43 | 12% | (5) | 56% | (24) | 33% | (14) | No | (Majority) |
| - | Compromised by the use of delayed fat grafting | 42 | 0% | (0) | 86% | (36) | 14% | (6) | No | (Consensus) |
| - | Depending on the use of systemic treatment | 34 | 38% | (13) | 53% | (18) | 9% | (3) | No | (Majority) |
|  | | | | | | | | | | |
| **NSM can be performed** | | | | | | | | | | |
| - | For any tumor size that does not involve the skin or nipple areola complex independent from axillary status | 41 | 80% | (33) | 12% | (5) | 7% | (3) | Yes | (Consensus) |
| - | For locally advanced breast cancer without the use of neoadjuvant chemotherapy | 43 | 44% | (19) | 44% | (19) | 12% | (5) | No consensus found | |
| - | For early breast cancer | 44 | 93% | (41) | 5% | (2) | 2% | (1) | Yes | (Consensus) |
| - | For DCIS | 42 | 93% | (39) | 0% | (0) | 7% | (3) | Yes | (Consensus) |
| - | In the risk reducing setting | 43 | 100% | (43) | 0% | (0) | 0% | (0) | Yes | (Consensus) |
| - | Only by specialized surgeons with sufficient  expertise in the technique | 42 | 90% | (38) | 7% | (3) | 2% | (1) | Yes | (Consensus) |

* Consensus is defined as votes above 75%, majority is defined as votes between 51–75%

Percentages may not total 100 due to rounding

**Supplementary appendix 5. Consensus conference results: Surgical technique**

|  | | **No. of responses** | **Percentage votes** | | | | | | **Voting result*** | |
| --- | --- | --- | --- | --- | --- | --- | --- | --- | --- | --- |
|  |  |  | **Yes (n)** | | **No (n)** | | **Abstain (n)** | |  | |
| **The risk of severe mastectomy flap necrosis requiring re-operation depends on the following surgical conditions or measures** | | | | | | | | | | |
| - | Location of the incision | 42 | 86% | (36) | 12% | (5) | 2% | (1) | Yes | (Consensus) |
| - | Surgical technology used for dissection (scalpel/scissors vs electrocautery vs PlasmaBlade™) | 44 | 36% | (16) | 48% | (21) | 16% | (7) | No consensus found | |
| - | Amount and duration of pressure applied by the retractors | 44 | 82% | (36) | 7% | (3) | 11% | (5) | Yes | (Consensus) |
| - | Thickness of the skin flaps | 44 | 84% | (37) | 9% | (4) | 7% | (3) | Yes | (Consensus) |
| - | Skin flap viability assessment (e.g., indocyanine green fluorescence, thermography) and re-excision of non-perfused skin during initial surgery | 43 | 16% | (7) | 37% | (16) | 47% | (20) | No consensus found | |
| - | Tissue pre-conditioning ± tissue post-conditioning ± local application of nitroglycerin cream | 41 | 10% | (4) | 39% | (16) | 51% | (21) | No consensus found | |
| - | Immediate use of compression bra or compression dressing | 41 | 22% | (9) | 51% | (21) | 27% | (11) | No | (Majority) |
| - | Technical expertise and experience of the surgeon | 42 | 93% | (39) | 5% | (2) | 2% | (1) | Yes | (Consensus) |
|  | | | | | | | | | | |
| **How thick should the mastectomy skin flap be? (please choose one of the following)** | | | | | | | | | | |
| - | Very thin, with dissection in the subdermal plane | 3 | 7% | (3) | . | . | . | . | . | |
| - | Thick, with dissection under the superficial fascia plane | 0 | 0% | (0) | . | . | . | . | . | |
| - | Thickness determined by the place / depth of the superficial fascia and dissection should follow this plane | 37 | 90% | (37) | . | . | . | . | . | |
| - | Abstain | 1 | 2% | (1) | . | . | . | . | . | |
| → Thickness determined by the place / depth of the superficial fascia and dissection should follow this plane (Consensus) | | | | | | | | | | |
|  | | | | | | | | | | |
| **Inframammary fold incision for NSM can be used** | | | | | | | | | | |
| - | Independent of breast size and shape | 36 | 8% | (3) | 83% | (30) | 8% | (3) | No | (Consensus) |
| - | For tumors with a distance < 1cm to the nipple on imaging | 42 | 43% | (18) | 43% | (18) | 14% | (6) | No consensus found | |
| - | For tumors in the upper inner quadrants in large breasts (≥ cup size C) | 40 | 33% | (13) | 58% | (23) | 10% | (4) | No | (Majority) |
| - | Independent of tumor size / location or breast size as long as the whole breast tissue is safely reached / removed and operative visibility is maintained | 43 | 72% | (31) | 26% | (11) | 2% | (1) | Yes | (Majority) |
|  | | | | | | | | | | |
| **Before inframammary fold is incised for NSM, a separate peri-areolar incision is recommended for retroareolar frozen section (please choose one of the following)** | | | | | | | | | | |
| - | Always | 1 | 3% | (1) | . | . | . | . | . | |
| - | Never | 27 | 68% | (27) | . | . | . | . | . | |
| - | For tumors with a distance < 1cm to the nipple on imaging | 9 | 23% | (9) | . | . | . | . | . | |
| - | Abstain | 3 | 8% | (3) | . | . | . | . | . | |
| → Never (Majority) | | | | | | | | | | |

* Consensus is defined as votes above 75%, majority is defined as votes between 51–75%

Percentages may not total 100 due to rounding

**Supplementary appendix 6. Consensus conference results: Contraindications**

|  | | **No. of responses** | **Percentage votes** | | | | | | **Voting result*** | |
| --- | --- | --- | --- | --- | --- | --- | --- | --- | --- | --- |
|  |  |  | **Yes (n)** | | **No (n)** | | **Abstain (n)** | |  | |
| **Outside of clinical trials, oncological contraindications to the preservation of the skin envelope are** | | | | | | | | | | |
| - | cT4b and cT4c breast cancer limited to a 1×1cm area of skin ulceration | 43 | 51% | (22) | 30% | (13) | 19% | (8) | Yes | (Majority) |
| - | Edema of skin envelope with enhancement on MRI  without redness | 42 | 71% | (30) | 19% | (8) | 10% | (4) | Yes | (Majority) |
| - | Absence of any distance between tumor and skin on preoperative imaging, but without clinical signs of skin infiltration | 40 | 50% | (20) | 43% | (17) | 8% | (3) | No consensus found | |
| - | Histological margin of less than 1mm on frozen section or definitive histology | 41 | 37% | (15) | 56% | (23) | 7% | (3) | No | (Majority) |
|  | | | | | | | | | | |
| **Outside of clinical trials, oncological contraindications to preservation of the nipple are** | | | | | | | | | | |
| - | Clinical signs of nipple involvement | 38 | 92% | (35) | 3% | (1) | 5% | (2) | Yes | (Consensus) |
| - | Any DCIS or invasive cancer in the margin to the nipple on histopathology (R1) | 38 | 89% | (34) | 5% | (2) | 5% | (2) | Yes | (Consensus) |
| - | Atypia in the margin to the nipple on frozen section, not 100% consistent with the diagnosis of DCIS or invasive cancer | 42 | 38% | (16) | 45% | (19) | 17% | (7) | No consensus found | |
| - | Distance between tumor and skin of nipple < 1cm on imaging or by palpation before surgery | 31 | 23% | (7) | 68% | (21) | 10% | (3) | No | (Majority) |
| - | Tumor ≥ 3cm in largest diameter | 40 | 3% | (1) | 93% | (37) | 5% | (2) | No | (Consensus) |
| - | Node positive breast cancer | 42 | 0% | (0) | 100% | (42) | 0% | (0) | No | (Consensus) |
| - | Carriers of mutations in BRCA 1 or BRCA 2 genes in the therapeutic or risk reducing setting | 39 | 0% | (0) | 97% | (38) | 3% | (1) | No | (Consensus) |
| - | Bloody nipple discharge | 37 | 54% | (20) | 35% | (13) | 11% | (4) | Yes | (Majority) |
| - | Clear nipple discharge | 37 | 14% | (5) | 65% | (24) | 22% | (8) | No | (Majority) |
|  | | | | | | | | | | |
| **In patients with a positive retroareolar margin** | | | | | | | | | | |
| - | Nipple may be excised without areola | 38 | 47% | (18) | 45% | (17) | 8% | (3) | No consensus found | |
| - | Adjuvant whole breast irradiation or intraoperative radiotherapy may suffice without excising nipple and areola | 41 | 29% | (12) | 59% | (24) | 12% | (5) | No | (Majority) |
|  | | | | | | | | | | |
| **In women with cup size ≥ C and ptosis ≥ Grade 2, but no other risk factors for nipple necrosis, NSM can be performed  with the use of** | | | | | | | | | | |
| - | Skin reduction and nipple-areola pedicles independent of the breast reconstruction technique | 39 | 79% | (31) | 10% | (4) | 10% | (4) | No | (Consensus) |
| - | Skin reduction and nipple-areola pedicles only when autologous breast reconstruction is planned | 38 | 11% | (4) | 84% | (32) | 5% | (2) | No | (Consensus) |
| - | Skin reduction and free nipple grafting | 41 | 78% | (32) | 7% | (3) | 15% | (6) | No | (Consensus) |
| - | Delayed timing after reduction mammoplasty | 40 | 70% | (28) | 8% | (3) | 23% | (9) | Yes | (Majority) |

* Consensus is defined as votes above 75%, majority is defined as votes between 51–75%

Percentages may not total 100 due to rounding

**Supplementary appendix 7. Consensus conference results: Special considerations in the risk reducing and therapeutic setting**

|  | | **No. of responses** | **Percentage votes** | | | | | | **Voting result*** | |
| --- | --- | --- | --- | --- | --- | --- | --- | --- | --- | --- |
|  |  |  | **Yes (n)** | | **No (n)** | | **Abstain (n)** | |  | |
| **During risk reducing NSM in carriers of mutations in BRCA 1 or BRCA 2 genes, removal of the sentinel nodes is  (please choose one of the following)** | | | | | | | | | | |
| - | Always recommended | 2 | 5% | (2) | . | . | . | . | . | |
| - | Never recommended | 15 | 37% | (15) | . | . | . | . | . | |
| - | Not recommended if a preoperative MRI reveals no abnormal finding in the breast | 24 | 59% | (24) | . | . | . | . | . | |
| - | Abstain | 0 | 0% | (0) | . | . | . | . | . | |
| → Not recommended if a preoperative MRI reveals no abnormal finding in the breast (Majority) | | | | | | | | | | |
|  | | | | | | | | | | |
| **During risk reducing NSM in carriers of mutations in BRCA 1 or BRCA 2 genes** | | | | | | | | | | |
| - | The removal of the pectoral fascia is recommended | 36 | 22% | (8) | 78% | (28) | 0% | (0) | No | (Consensus) |
| - | The removal of the infra-mammary fold is recommended | 42 | 10% | (4) | 90% | (38) | 0% | (0) | No | (Consensus) |
| - | The soft tissue should be removed all the way to the clavicle | 37 | 8% | (3) | 89% | (33) | 3% | (1) | No | (Consensus) |
| - | Routine retro-areolar frozen section analysis is recommended | 41 | 12% | (5) | 80% | (33) | 7% | (3) | No | (Consensus) |
| - | The breast glandular tissue should be removed according to its anatomical borders | 39 | 92% | (36) | 8% | (3) | 0% | (0) | Yes | (Consensus) |
|  | | | | | | | | | | |
| **Routinely, during therapeutic NSM in patients with breast cancer** | | | | | | | | | | |
| - | The removal of the pectoral fascia is recommended | 34 | 47% | (16) | 50% | (17) | 3% | (1) | No consensus found | |
| - | The removal of the infra-mammary fold is recommended | 40 | 13% | (5) | 85% | (34) | 3% | (1) | No | (Consensus) |
| - | The soft tissue should be removed all the way to the clavicle | 38 | 16% | (6) | 82% | (31) | 3% | (1) | No | (Consensus) |
| - | Routine retro-areolar frozen section analysis is recommended | 38 | 42% | (16) | 55% | (21) | 3% | (1) | No | (Majority) |
| - | The breast glandular tissue should be removed according to its anatomical borders | 29 | 93% | (27) | 3% | (1) | 3% | (1) | Yes | (Consensus) |

* Consensus is defined as votes above 75%, majority is defined as votes between 51–75%

Percentages may not total 100 due to rounding

**Supplementary appendix 8. Consensus conference results:** **Breast reconstruction**

|  | | **No. of responses** | **Percentage votes** | | | | | | **Voting result*** | |
| --- | --- | --- | --- | --- | --- | --- | --- | --- | --- | --- |
|  |  |  | **Yes (n)** | | **No (n)** | | **Abstain (n)** | |  | |
| **In your routine clinical practice, there are established indications for the use of** | | | | | | | | | | |
| - | Synthetic meshes | 41 | 32% | (13) | 56% | (23) | 12% | (5) | No | (Majority) |
| - | Acellular dermal matrix | 39 | 49% | (19) | 44% | (17) | 8% | (3) | No consensus found | |
|  | | | | | | | | | | |
| **In a non-obese woman with cup A sized breasts and no preference concerning type of reconstruction and no planned post-mastectomy radiotherapy, your preferred method of breast reconstruction after NSM is (please choose one of the following)** | | | | | | | | | | |
| - | Immediate autologous reconstruction | 6 | 15% | (6) | . | . | . | . | . | |
| - | Immediate one-stage pre-pectoral implant-based reconstruction without synthetic mesh or acellular dermal matrix | 1 | 3% | (1) | . | . | . | . | . | |
| - | Immediate one-stage sub-pectoral implant-based reconstruction without synthetic mesh or acellular dermal matrix | 14 | 35% | (14) | . | . | . | . | . | |
| - | Immediate one-stage pre-pectoral implant-based reconstruction with synthetic mesh | 1 | 3% | (1) | . | . | . | . | . | |
| - | Immediate one-stage sub-pectoral implant-based reconstruction with synthetic mesh | 4 | 10% | (4) | . | . | . | . | . | |
| - | Immediate one-stage pre-pectoral implant-based reconstruction with acellular dermal matrix | 5 | 13% | (5) | . | . | . | . | . | |
| - | Immediate one-stage sub-pectoral implant-based re- construction augmented with acellular dermal matrix | 6 | 15% | (6) | . | . | . | . | . | |
| - | Two-stage implant-based reconstruction (sub-pectoral expander to definite implant) | 1 | 3% | (1) | . | . | . | . | . | |
| - | Abstain | 2 | 5% | (2) | . | . | . | . | . | |
| → No consensus found | | | | | | | | | | |

* Consensus is defined as votes above 75%, majority is defined as votes between 51–75%

Percentages may not total 100 due to rounding

**Supplementary appendix 9. Consensus conference results:** **Registries and outcome assessment**

|  | | **No. of responses** | **Percentage votes** | | | | | | **Voting result*** | |
| --- | --- | --- | --- | --- | --- | --- | --- | --- | --- | --- |
|  |  |  | **Yes (n)** | | **No (n)** | | **Abstain (n)** | |  | |
| **Patients undergoing NSM should be included in** | | | | | | | | | | |
| - | National and/or international prospective registries (such as INSPIRE) | 39 | 90% | (35) | 10% | (4) | 0% | (0) | Yes | (Consensus) |
|  | | | | | | | | | | |
| **Outcome assessment of NSM should be standardized to include** | | | | | | | | | | |
| - | Pre- and postoperative pictures | 40 | 95% | (38) | 0% | (0) | 5% | (2) | Yes | (Consensus) |
| - | Patient-reported outcome measurements | 39 | 77% | (30) | 18% | (7) | 5% | (2) | Yes | (Consensus) |
| - | The EORTC QLQ-BRECON23 quality-of-life questionnaire | 37 | 22% | (8) | 30% | (11) | 49% | (18) | No consensus found | |
| - | All scales of the BREAST-Q – reconstruction module version 2.0 | 40 | 15% | (6) | 65% | (26) | 20% | (8) | No | (Majority) |
| - | Selected scales of the BREAST-Q – reconstruction module version 2.0 (e.g., satisfaction with breasts scale +/- psychosocial well-being) | 42 | 69% | (29) | 19% | (8) | 12% | (5) | No | (Majority) |

* Consensus is defined as votes above 75%, majority is defined as votes between 51–75%

Percentages may not total 100 due to rounding
